# Exoskeletons of Bougainvilliidae and other Hydroidolina (Cnidaria, Hydrozoa): structure and composition

**DOI:** 10.7717/peerj.2964

**Published:** 2017-02-16

**Authors:** María A. Mendoza-Becerril, José Eduardo A.R. Marian, Alvaro Esteves Migotto, Antonio Carlos Marques

**Affiliations:** 1 Department of Zoology, Institute of Biosciences, University of São Paulo, São Paulo, Brazil; 2 Center for Marine Biology, University of São Paulo, São Sebastião, São Paulo, Brazil

**Keywords:** “Anthoathecata”, Exosarc, Histochemistry, Perisarc, Leptothecata

## Abstract

The exoskeleton is an important source of characters for the taxonomy of Hydroidolina. It originates as epidermal secretions and, among other functions, protects the coenosarc of the polypoid stage. However, comparative studies on the exoskeletal tissue origin, development, chemical, and structural characteristics, as well as its evolution and homology, are few and fragmented. This study compares the structure and composition of the exoskeleton and underlying coenosarc in members of “Anthoathecata” and some Leptothecata, but does so mainly in bougainvilliid polyps histological analyses. We also studied the development of the exoskeleton under experimental conditions. We identified three types of glandular epidermal cells related to the origin of the exoskeleton and the secretion of its polysaccharides component. The exoskeleton of the species studied is either bilayered (perisarc and exosarc, especially in bougainvilliids) or corneous (perisarc). The exoskeleton varies in chemical composition, structural rigidity, thickness, extension, and coverage in the different regions of the colony. In bilayered exoskeletons, the exosarc is produced first and appears to be a key step in the formation of the rigid exoskeleton. The exoskeleton contains anchoring structures such as desmocytes and “perisarc extensions.”

## Introduction

The exoskeleton in Hydroidolina originates as epidermal secretions (Knight, 1970; Sorauf, 1980; Kossevitch, Herrmann & Berking, 2001). The epithelial epidermal layer of the living tissue, coenosarc, of benthic colonial or solitary polyps is composed of diverse cell types (e.g., epitheliomuscular, interstitial, glandular, nervous and cnidocytes; Chapman, 1974; Mackie, 1984).

The glandular epithelial cells of Hydroidolina are responsible for secreting compounds (e.g., structural proteins and enzymes, phenols, polysaccharides) that are associated with the development of the exoskeleton (Knight, 1970; Kossevitch, Herrmann & Berking, 2001; Böttger et al., 2012; Hwang et al., 2013; Mendoza-Becerril et al., 2016). These compounds have been identified in other organisms; for example, in the chitin in fungi, the chitin can have different morphological expression or it cannot be expressed (Wagner, 1994). The chitin system of the exoskeleton can be split into molecular matrix (MM) and molecular synthesis (MSS) (Wagner, 1994). The MM is the extracellular substance containing the molecules, and is located at the outer surface of the epithelium, while the MSS is the biosynthetic apparatus that produces the genetically encoded molecules (Wagner, 1994).

The exoskeleton is laid down in the growing places where colonial elements are developed, such as stolon and internodes, hydranth, and growing tips (Kosevich, 2013), participating in biological aspects such as protection and flexibility (cf. Mendoza-Becerril et al., 2016). Trophosome exoskeleton has been considered a key morphological diagnostic character at different taxonomic levels for Hydroidolina (Cornelius, 1995; Schuchert, 2012), although some characteristics, such as the thickness and presence of annulations, and the development of hydrotheca, gonotheca and nematotheca, are taxonomically more relevant for Leptothecata (e.g., Cornelius, 1982; Cornelius, 1995; Cunha, Genzano & Marques, 2015) than for “Anthoathecata” (a non-monophyletic group (e.g., Collins et al., 2006; Cartwright et al., 2008; Van Iten et al., 2014).

Little is known about the nature of the exoskeleton in Hydroidolina, and few studies have investigated its origin, structure, and composition (e.g., Bouillon, 1966). The majority of these studies have focused on Leptothecata (e.g., Berrill, 1950; Knight, 1970; Kossevitch, Herrmann & Berking, 2001; Tretenichenko et al., 2006; Kosevich, 2013; Hwang et al., 2013), with scattered studies for “Anthoathecata” (e.g., Congdon, 1906; Berrill, 1949; Cowden, 1965; Wineera, 1968; Wineera, 1972; Yamada et al., 2007; Mendoza-Becerril et al., 2016). The exoskeleton also provides important characters at various taxonomic levels of “Anthoathecata” in general (e.g., Bougainvilliidae (Allman, 1864; Petersen, 1979; Calder, 1988; Schuchert, 2007); Milleporidae (Razak & Hoeksema, 2003); Stylasteridae (Cairns, 2011); and Hydractiniidae (Miglietta, McNally & Cunningham, 2010))

“Anthoathecata” has the widest exoskeletal variability and structural complexity among Hydrozoa, encompassing corneous, coriaceous or bilayered exoskeletons (Mendoza-Becerril et al., 2016). Bilayered exoskeletons, such as those of the pseudo-hydrotheca, common in Bougainvilliidae (“Filifera”), are formed by a corneous chitin-protein reinforced by a covering exosarc formed by glycosaminoglycans (GAGs) with incrusting inorganic and/or organic particles (Mendoza-Becerril et al., 2016). Morphological, histological and histochemical studies of bilayered exoskeletons are needed in order to understand the evolution and homology of these structures, as well as their function.

The aim of this study is to use histological and histochemical observations to analyzed and compare the structure and composition of the coenosarc and exoskeleton in polyps of five families of “Anthoathecata” and three families of Leptothecata, focusing on variable exoskeleton of the poorly known Bougainvilliidae Lütken, 1850 (Hydroidolina, “Anthoathecata”). Additionally, we investigated the formation of the exoskeleton under different experimental conditions for five species of Bougainvilliidae, Pandeidae, and Oceaniidae, to verify the presence of MM and understand its cellular origin, morphology and variation in chemical compositional.

## Material and Methods

### Taxa sampled and histology

Samples included specimens from the collection of the Museum of Zoology, University of São Paulo (MZUSP); Laboratory of Marine Evolution, Institute of Biosciences, University of São Paulo (LEM-IBUSP); Nagera Station, University of Mar del Plata, Argentina (UNMdP); and the National Museum of Natural History, Smithsonian Institution (USNM) (Table 1). Materials were fixed either in 4% formaldehyde solution in seawater or in 92% ethanol. Samples were dehydrated and embedded in glycol methacrylate resin (Leica Historesin Embedding Kit; Leica Microsystems Nussloch GmbH, Germany). Serial longitudinal sections (3 to 7 µm) (the few exceptions in transverse sections are noted in the figure captions) were stained with toluidine blue (TB), hematoxylin-hosin (HE), periodic acid-Schiff (PAS, for identification of polysaccharides—P), alcian blue pH 2.5 (AB, for identification of glycosaminoglycans—GAGs), mercury-bromophenol blue (HgBpB, for identification of proteins), and naphthol yellow S (NYS, for identification of proteins) (McManus, 1946; Deitch, 1955; Mowry, 1963; Pearse, 1985) (Table 2). Whenever possible we measured the thickness of each exoskeletal layer of the hydrorhiza, hydrocaulus, hydranth and gonophore. Histological slides are deposited in the collection of the Laboratory of Marine Evolution, Institute of Biosciences, University of São Paulo and the National Museum of Natural History, Smithsonian Institution. Voucher specimens are deposited in the Museum of Zoology, University of São Paulo (Table 1), and the National Museum of Natural History, Smithsonian Institution (Table 1). The classification adopted in the study follows Collins et al. (2006), Cartwright et al. (2008), Maronna et al. (2016), an Cunha, Collins & Marques (2017), representing the most updated framework available for the taxonomy of hydrozoans.

**Table 1:** Sampling sites and museum vouchers for the hydroidolinan species included in the histological and culture analyses.

Material examined	Sampling site	Coordinates		Data	Substrate	Gonophore	Depth (m)	Collector	Museum voucher	Culture conditions/ Histology
**Phylum Cnidaria Verrill, 1865**										
**Subphylum Medusozoa** **Petersen, 1979**										
**Class Hydrozoa Owen 1843**										
**Subclass Hydroidolina Collins 2000**										
**Superorder “Anthoathecata”** **Cornelius, 1992**										
**Order Capitata Kühn, 1913** ***sensu stricto***										
**Family Pennariidae McCrady, 1859**										
*Pennaria disticha* Goldfuss, 1820	Brazil, São Paulo, São Sebastião, Pitangueiras Beach	23°49.48′S	045°25.19′W	23/09/2013	Rock	Absent	1	MA Mendoza-Becerril	LEM-IBUSP_7	−∕ +
**Family Bougainvilliidae Lütken, 1850**										
*Bimeria vestita* Wright, 1859	Brazil, São Paulo, São Sebastião, Yacht Club Ilhabela	23°46.37′S	045°21.35′W	2013	Shell	Absent	<1	MA Mendoza-Becerril	LEM-IBUSP_1	+∕ +
	Brazil, Paraná, Paranaguá, Ilha do Mel	25°34.00′S	048°18.00′W	02/1997	Octocoral *Carijoa riisei*	Present	–	MA Haddad	MZUSP 5201	−∕ +
	Argentina, Mar del Plata	38°4.55′S	057°32.32′W	10/08/1990	–	Absent	Intertidal	–	UNMdP Hd3-38	−∕ +
*Bougainvillia muscus* (Allman, 1863)	Brazil, São Paulo, São Sebastião, Yacht Club Ilhabela	23°46.37′S	045°21.35′W	–	–	–	–	–	LEM-IBUSP_2	−∕ +
	Brazil, São Paulo, Segredo Beach	23°49.68′S	045°25.36′W	2013	Shell and artificial	Absent	<1	MA Mendoza-Becerril	LEM-IBUSP_3	+∕ +
	Brazil, Santa Catarina, Bombinhas, Tainha Beach	27°12.97′S	048°30.61′W	02/12/2006	Hydrozoa *Eudendrium* sp.	Present	–	AC Marques and E Ale	MZUSP 4217	−∕ +
	Argentina, Comodoro Rivadavia	45°52.93′S	067°29.06′W	01/2013	–	Absent	–	–	UNMdP Hd11-128	−∕ +
*Bougainvillia rugosa* Clarke, 1882	Brazil, Santa Catarina, Penha, Enseada da Armação do Itaporoy	26°46.26′S	048°36.48′W	24/06/2005	*Perna perna*	Absent	2	EC Bornancin	MZUSP 4332	−∕ +
*Bougainvillia* sp.	USA, California, San Pedro, Duffey’s Float	33°41.72′N	118°18.47′W	30/12/1901	–	Present	–	–	USNM 43497	−∕ +
*Dicoryne conferta* Alder, 1856	USA, Massachusetts, Gloucester Harbor	42°35.43′N	070°40.40′W	16/08/1878	Mollusca	Present	110	United States Fish Commission	USNM 20234	−∕ +
	Canada, Newfoundland, south of Peter’s Bank	47°19.45′N	056°46.91′W	04/06/2005	–	Present	368	–	USNM 43967	−∕ +
*Garveia annulata* Nutting, 1901	USA, California, Monterey Bay, Carmel Point	36°32.31′N	122°0.77′W	31/10/1978	–	Present	Intertidal	J Cooper	USNM 71026	−∕ +
*Garveia franciscana* (Torrey, 1902)	USA, California, Martinez	38°0.99′N	122°24.71′W	–	–	Present	–	–	USNM 43496	−∕ +
	Panama, Gulf of Panama	8°11.26′N	079°33.42′W	26/08/1974	–	Present	–	–	USNM 89229	−∕ +
*Garveia gracilis* (Clark, 1876)	Panama, Gulf of Panama	8°11.26′N	079°33.42′W	29/03/1973		Absent		S Hildebrand	USNM 43330	−∕ +
*Garveia nutans* Wright, 1859	United Kingdom, England, Plymouth Sound,	50°20.77′N	004°8.87′W	–		Present	–	GE Bullen	USNM 29449	−∕ +
*Pachycordyle michaeli* (Berril, 1948)	USA, Maine, Port Harbor Marine	43°38.46′S	070°13.34′W	28/07/2007	Rock	Present		AC Marques	MZUSP 1832	−∕ +
*Parawrightia robusta* Warren, 1907	Brazil, Pará, Atalaia Beach	00°35.60′S	047°18.71′W	04/07/2012	Rock	Absent	Intertidal	AF Cunha and MA Mendoza-Becerril	MZUSP 003390	−∕ +
	Brazil, São Paulo, São Sebastião, Yacht Club Ilhabela	23°46.37′S	045°21.35′W	2013	Ascidian	Absent	<1	MA Mendoza-Becerril	LEM-IBUSP_4	+∕ +
	Brazil, Santa Catarina, Itapoá	26°07.016′S	048°36.967′W	25/10/2003	Ascidian	Absent	Intertidial	MA Haddad	MZUSP 4379	−∕ +
*Rhizorhagium* sp.	USA, Washington, Puget Sound	47°42.05′N	122°28.18′W	–	–	Absent	–	–	USNM 42339	−∕ +
**Family Eudendriidae L. Agassiz, 1862**										
*Eudendrium carneum* Clarke, 1882	Brazil, Alagoas, Barra de São Miguel	09°50.00′S	035°53.08′W	22/10/2006	–	Absent	0–3	AC Marques	MZUSP 1673	−∕ +
**Family Oceaniidae Eschscholtz, 1829**										
*Turritopsis* sp.	Brazil, São Paulo, São Sebastião, Segredo Beach	23°49.68′S	045°25.36′W	03/11/2013	Shell	Absent	<1	MA Mendoza-Becerril	LEM-IBUSP_5	+∕ +
**Family Pandeidae Haeckel, 1879**										
*Leuckartiara* cf. *octona* (Fleming, 1823)	Brazil, São Paulo, São Sebastião, Segredo Beach	23°49.68′S	045°25.36′W	13/11/2013	Shell *Strombus pugilis*	Absent	4	JM Oliveira and AAW Monteiro	LEM-IBUSP_6	+∕ +
**Superorder Leptothecata Cornelius, 1992**										
**Order Macrocolonia Leclère et al., 2009**										
**Suborder Haleciida Bouillon, 1984** ***sensu* Maronna et al., 2016**										
**Family Haleciidae Hincks, 1868**										
*Halecium bermudense* Congdon, 1907	Brazil, São Paulo, São Sebastião	23°45.17′S	045°24.41′W	08/2010	Artificial	Present	Surface	M Fernandez	MZUSP 003391	−∕ +
**Order Statocysta Leclère et al., 2009**										
**Suborder Proboscoida Broch, 1910** ***sensu* Maronna et al., 2016 and Cunha, Collins & Marques, 2017**										
**Infraorder Obeliida** **Maronna et al., 2016**										
**Family Clytiidae Cockerell, 1911**										
*Clytia gracilis* (M. Sars, 1850)	Brazil, Santa Catarina, Bombinhas, Tainha Beach	27°12.97′S	048°30.61′W	02/12/2006	Hydrozoa *Eudendrium carneum*	Present	5–7	AC Marques and E Ale	MZUSP 4210	−∕ +
*Orthopyxis sargassicola* (Nutting, 1915)	Brazil, Alagoas, Maceió	09°40.84′S	035°42.67′W	25/10/2006	–	Present	0–2	AC Marques	MZUSP 1740	−∕ +
**Family Obeliidae** **Maronna et al., 2016**										
*Obelia dichotoma* (Linnaeus, 1758)	Brazil, Pará, Salinópolis, Farol Velho Beach	00°35.46′S	047°19.48′W	03/07/2012	Rock	Present	Surface	AF Cunha and MA Mendoza-Becerril	MZUSP 3371	−∕ +
	Slovenia, Piran, Farol Amarelo	45°35.66′N	013°42.28′E	01/08/2011	–	Absent	–	AC Morandini and LS Miranda	MZUSP 3369	−∕ +
	USA, Massachusetts, Westport	41°30.21′N	076°3.77′W	26/07/2010	–	Absent	–	AC Marques	LEM-IBUSP_8	−∕ +

**Notes.**

MZUSP Museum of Zoology, University of São Paulo LEM-IBUSP Laboratory of Marine Evolution, Institute of Biosciences, University of São Paulo UNMdP Nagera Station, University of Mar del Plata, Argentina USNM the National Museum of Natural History, Smithsonian Institution – data not available + analized

**Table 2:** Staining methods times in hystoresin to histological analyses.

Staining method	Sections (µm)	Reagents	Time	Wash (times)	Temperature (°C)
Toluidine blue	3	Toluidine blue	0:01:30	–	room
		Destilled water	–	1	room
Hematoxylin-Eosin	3	Hematoxylin	0:15:00	–	39
		Tap water	0:03:00	–	room
		Destilled water	–	1	room
		Eosina	0:05:00	–	39
		Destilled water	0:02:00	3	room
		Alcohol 70%	–	1	room
		Destilled water	–	1	room
Periodic acid-Schiff	5–7	Periodic acid	0:20:00	–	39
		Destilled water	0:02:00	3	room
		Schiff	1:00:00	–	room
		Tap water	0:05:00	–	room
		Destilled water	–	1	room
Alcian blue pH 2.5	5–7	Acetic acid 3%	0:06:00		room
		Alcian blue pH 2.5	2:00:00	–	39
		Tap water	0:03:00		room
		Destilled water	–	1	room
Mercury-bromophenol blue	5–7	Mercury-bromophenol blue	1:00:00	–	room
		Acetic acid 0.5%	0:10:00	–	room
		Buffer solution	–	1	room
Naphthol yellow S	5–7	Naphthol yellow S	1:00:00	–	room
		Acetic acid 1.0%	0:10:00	–	room
		Tertiary butyl alcohol	–	1	room

**Notes.**

–, not applicable data.

### Culture of colonies

Infertile colonies of *Bimeria vestita*, *Bougainvillia muscus, Leuckartiara* cf. *octona*, *Parawrightia robusta, Turritopsis* sp. and *Clytia* sp. were collected from the intertidal zone in São Sebastião, São Paulo State, Brazil. The colonies were carefully cut into small pieces and maintained on glass plates in plastic boxes containing aerated seawater at room temperature (22.3 ± 1.4 °C) with artificial lighting (15–16 h light, 9–8 h dark). Seawater was changed every three days, and animals were fed twice a day with plankton or nauplii of *Artemia salina*.

We investigated the development of the exoskeleton and autogenous or exogenous (e.g., produced by aggregated microorganisms such as diatoms) of the exosarc by maintaining the colonies under two different conditions (for approximately one week): A, with unfiltered seawater (21.9 ± 0.8 °C); and B, with filtered seawater (22.2 ± 0.6 °C). In the latter experiment, the seawater was filtered using a <25 µm filter, and the animals were fed individually in small finger-bowls to avoid contamination with organic and inorganic particles. In this experiment, the seawater was changed and the glass plates cleaned daily.

## Results

**Figure 1:**
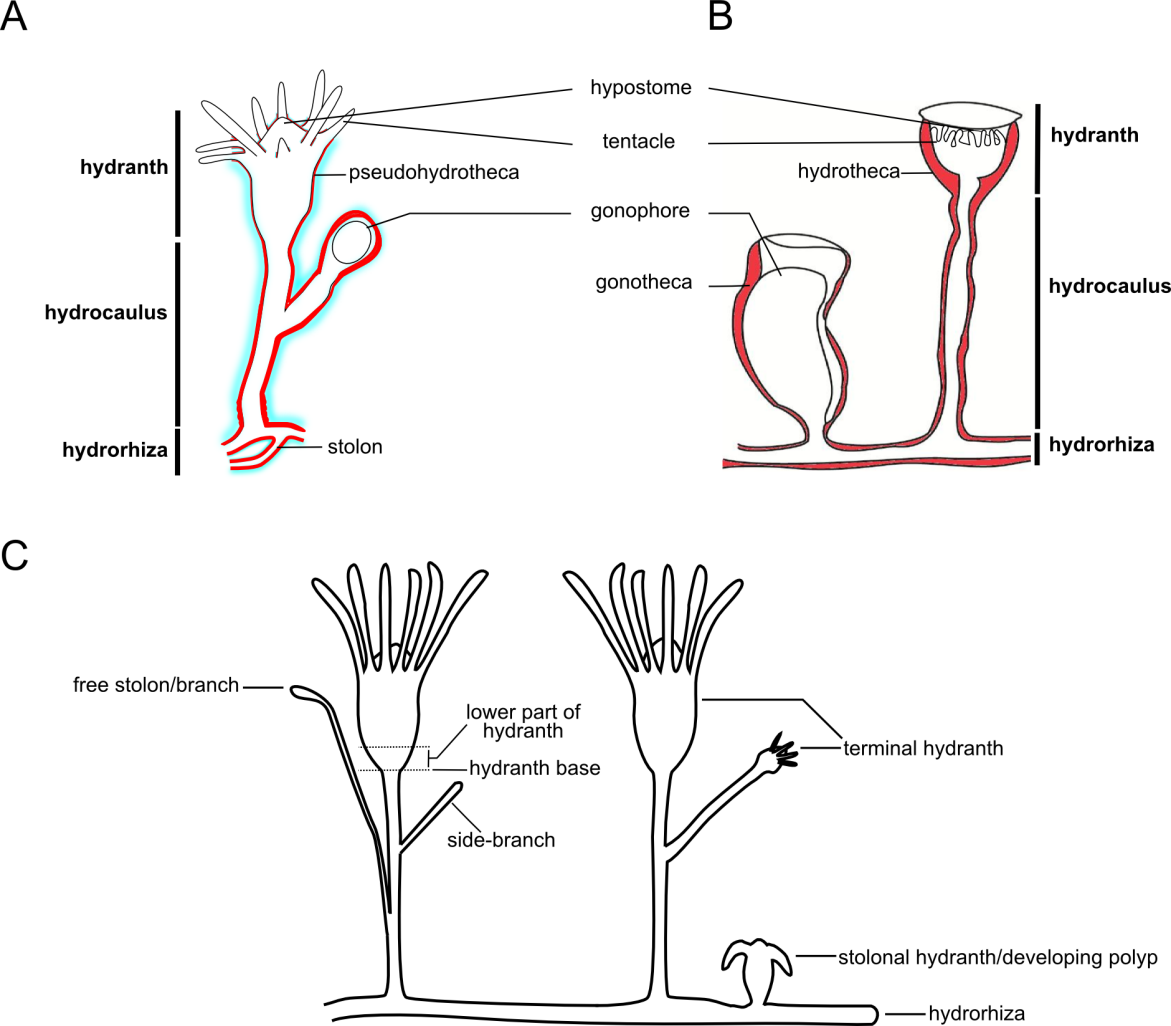
Anatomy of polyps. (A) Polyp of “Anthoathecata”; (B) polyp of Leptothecata; (C) main regions described for the exoskeleton.

The analysis of the longitudinal sections revealed the presence of different patterns of organization. For the hydroids, there are three morphologically distinct patterns, *viz.*, the basal hydrorhiza (formed by stolons), median hydrocaulus (=stem, stalk), and distal hydranth (Figs. 1A–1B). On the other hand, early stages of colonial development (in culture conditions) had five different patterns of organization, *viz.*, the free stolon/branch, hydrorhiza, side-branch, stolonal hydranth/developing polyp, and terminal hydranth (Fig. 1C).

**Figure 2:**
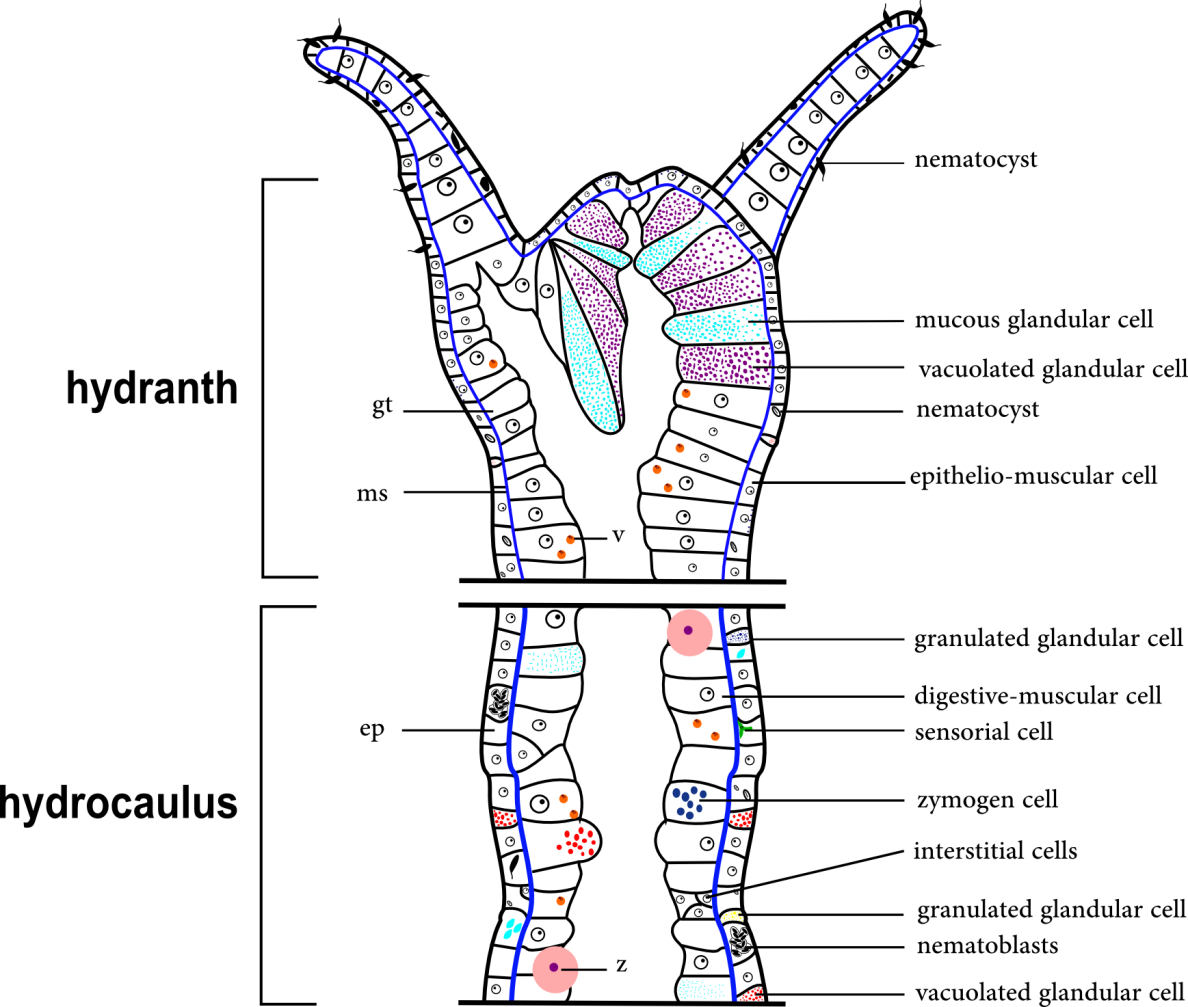
Schematic drawing of the coenosarc of a generalized polyp of Hydroidolina. Abbreviations: z, zooxanthellae; ep, epidermis; gt, gastrodermis; ms, mesoglea; v, vacuole.

The epidermal layer is thin in the region of the tentacles and gonophores. A thin, acellular mesoglea underlies the polyp epidermis and is thinner in the tentacles. The gastrodermis is a thick layer and contains some cells that most likely correspond to zooxanthellae (appears to be *Symbiodinium*), observed in only some species (e.g., *Halecium bermudense*) (Fig. 2).

We provide below histological/histochemical description of the exoskeleton in Bougainvilliidae and other members of Hydroidolina, as well as of the epidermal cells that may be associated with the exoskeleton and anchoring structures (Tables 3–6).

### Exoskeleton organization in Bougainvilliidae Lütken, 1850 and other “Anthoathecata”

Staining was variable among the different glandular cells. In general the bougainvilliid hydroids present three types of epidermal glandular cells: vacuolated glandular cells (PAS-positive cells), highly granulated glandular cells (HgBpB and NYS-positive cells), and mucous glandular cells (PAS, HgBpB, and AB-positive cells). In other “Anthoathecata,” such as *Eudendrium carneum* (Eudendriidae) we observed vacuolated glandular cells with affinity for TB and PAS and mucous glandular cells, positively for H, PAS and AB, while *Turritopsis* sp. (Oceaniidae) glandular cells with affinity for PAS, HgBpB and NYS. In the hydranth of *Leuckartiara* cf. *octona* (Pandeidae), we observed cells with thin granular film apically and granules. In some “Anthoathecata,” *Turritopsis* sp. and *L.* cf. *octona* i-cells are commonly found grouped at the base of the hydrocaulus epidermis.

**Table 3:** Reactions of the exoskeletal layers of several hydroidolinan species to specific staining.

Species	Layer	Region/staining	TB	HE	Schiff	PAS	AB	HgBpB	NYS
**“Anthoathecata”**									
**Pennariidae**									
*Pennaria disticha*	Inner layer	Hydrorhiza	+++ blue	+++ magenta	–	+++ magenta	–	+++ blue	+++ yellow
		Hydrocaulus	+++ blue	+++ magenta	–	+++ magenta	–	+++ blue	+++ yellow
		Hydranth	ø	ø	ø	ø	ø	ø	ø
	Outer layer	Hydrorhiza	+++ purple	+ magenta	<+ magenta	++ magenta	++ alcian blue	–	–
		Hydrocaulus	+++ purple	+ magenta	<+ magenta	++ magenta	++ alcian blue	–	–
		Hydranth	ø	ø	ø	ø	ø	ø	ø
**Bougainvilliidae**									
*Bimeria vestita*	Inner layer	Hydrorhiza	+++ blue	++ magenta	–	++ magenta	–	+++ blue	+++ yellow
		Hydrocaulus	+++ blue	++ magenta	–	++ magenta	–	+++ blue	+++ yellow
		Hydranth	+ blue	+ magenta	–	+ magenta	–	<+ blue	<+ yellow
		Gonophore	+ blue	+ magenta	–	+ magenta	–	<+ blue	<+ yellow
	Outer layer	Hydrorhiza	+++ purple	+ magenta	<+ magenta	+ magenta	++ alcian blue	–	–
		Hydrocaulus	+++ purple	++ magenta	<+ magenta	+ magenta	++ alcian blue	–	–
		Hydranth	+++ purple	++ magenta	<+ magenta	+ magenta	+++ alcian blue		
		Gonophore	+++ purple	+ magenta		+ magenta	++ alcian blue	–	–
*Bougainvillia muscus*	Inner layer	Hydrorhiza	+++ blue	++ magenta	–	+++ magenta	–	+++ blue	+++ yellow
		Hydrocaulus	+++ blue	++ magenta	–	+++ magenta	–	+++ blue	+++ yellow
		Hydranth	+ blue	<+ magenta	–	+ magenta	–	<+ blue	<+ yellow
		Gonophore	+ blue	+magenta	–	+ magenta	–	<+ blue	<+ yellow
	Outer layer	Hydrorhiza	+++ purple	++ magenta	<+ magenta	++ magenta	++ alcian blue	–	–
		Hydrocaulus	+++ purple	++ magenta	<+ magenta	++ magenta	++ alcian blue	–	–
		Hydranth	+++ purple	++ magenta	<+ magenta	++ magenta	+++ alcian blue	–	–
		Gonophore	+++ purple	+ magenta	–	++ magenta	++ alcian blue	–	–
*Bougainvillia rugosa*	Inner layer	Hydrorhiza	+ blue	+ magenta	–	+ magenta	–	++ blue	+++ yellow
		Hydrocaulus	++ blue	+ magenta	–	+ magenta	–	++ blue	+++ yellow
		Hydranth	+ blue	+ magenta	–	+ magenta	–	<+ blue	+ yellow
		Gonophore	+ blue	<+ magenta	–	++ magenta	–	<+ blue	<+ yellow
	Outer layer	Hydrorhiza	+++ purple	++ magenta	<+ magenta	+++ magenta	+++ alcian blue	–	–
		Hydrocaulus	+++ purple	++ magenta	<+ magenta	+++ magenta	+++ alcian blue	–	–
		Hydranth	+++ purple	++ magenta	<+ magenta	+++ magenta	+++ alcian blue	–	–
		Gonophore	+++ purple	++ magenta	–	++ magenta	++ alcian blue	–	–
*Bougainvillia* sp.	Inner layer	Hydrorhiza	x	x	x	x	x	x	x
		Hydrocaulus	+++ blue	+++ magenta	<+ magenta	+++ magenta	–	+++ blue	+++ yellow
		Hydranth	++ blue	+ magenta	<+ magenta	+ magenta	<+ alcian blue	<+ blue	–
		Gonophore	+ blue	+ magenta	<+ magenta	+ magenta	<+ alcian blue	<+ blue	–
	Outer layer	Hydrorhiza	x	x	x	x	x	x	x
		Hydrocaulus	+++ purple	+ magenta	–	++ magenta	+++ alcian blue	–	–
		Hydranth	+++ purple	+ magenta	–	++ magenta	+ alcian blue	–	–
		Gonophore	+++ purple	+ magenta	–	+++ magenta	+ alcian blue	–	–
*Dicoryne conferta*	Inner layer	Hydrorhiza	+++ blue	++ purple	+ magenta	++ magenta	<+ alcian blue	+ blue	+ yellow
		Hydrocaulus	+++ blue	++ purple	+ magenta	++ magenta	<+ alcian blue	+ blue	+ yellow
		Hydranth	+ blue	+ magenta	+ magenta	+ magenta	+ alcian blue	+ blue	<+ yellow
		Gonophore	+ blue	+ magenta	<+ magenta	++ magenta	+ alcian blue	+ blue	–
	Outer layer	Hydrorhiza	+++ purple	+ purple	<+ magenta	++ magenta	+++ alcian blue	–	+ brown
		Hydrocaulus	+++ purple	+ purple	<+ magenta	+++ magenta	+++ alcian blue	–	+ brown
		Hydranth	++purple	+ magenta	<+ magenta	+++ magenta	+++ alcian blue	–	–
		Gonophore	++purple	<+ magenta	<+ magenta	+ magenta	+++ alcian blue	–	–
*Garveia annulata*	Inner layer	Hydrorhiza	x	x	x	x	x	x	x
		Hydrocaulus	+++ blue	+++ magenta	–	+++ magenta	–	+++ blue	+++ yellow
		Hydranth	+++ blue	+++ magenta	–	+++ magenta	–	+++ blue	+++ yellow
		Gonophore	+++ blue	?	–	+++ magenta	–	+ blue	<+ yellow
	Outer layer	Hydrorhiza	x	x	x	x	x	x	x
		Hydrocaulus	++ purple	++ magenta	–	+++ magenta	++ alcian blue	–	–
		Hydranth	++ purple	++ magenta	–	++ magenta	++ alcian blue	–	–
		Gonophore	++ purple	++ magenta	–	+++ magenta	+ alcian blue	–	–
*Garveia franciscana*	Inner layer	Hydrorhiza	x	x	x	x	x	x	x
		Hydrocaulus	+ blue	++ magenta	<+ magenta	++ magenta	–	+++ blue	+++ yellow
		Hydranth	<+ blue	+ magenta	–	+ magenta	–	–	–
		Gonophore	+ blue	+ magenta	<+ magenta	+ magenta	–	+ blue	+ yellow
	Outer layer	Hydrorhiza	*	*	*	*	*	*	*
		side-branch	+++ purple	+++ magenta	<+ magenta	+++ magenta	+++ alcian blue	++ blue	–
		Hydranth	+++ purple	++ magenta	–	+++ magenta	+++ alcian blue	–	+ brown
		Gonophore	+++ purple	++ magenta	–	++ magenta	+++ alcian blue	–	+ brown
*Garveia gracilis*	Inner layer	Hydrorhiza	x	x	x	x	x	x	x
		Hydrocaulus	+++ blue	+++ magenta	–	+ magenta	–	+++ blue	+++ yellow
		Hydranth	+ blue	+ magenta	–	+ magenta	–	–	–
	Outer layer	Hydrorhiza	x	x	x	x	x	x	x
		Hydrocaulus	++ purple	<+ magenta	<+ magenta	+++ magenta	++ alcian blue	–	<+ brown
		Hydranth	+++ purple	+ magenta	+ magenta	+++ magenta	++ alcian blue	–	+ brown
*Garveia nutans*	Inner layer	Hydrorhiza	x	x	x	x	x	x	x
		Hydrocaulus	+++ blue	++ magenta	<+ magenta	+++ magenta	+++ alcian blue	+++ blue	++ yellow
		Hydranth	+ blue	++ magenta	–	+ magenta	+ alcian blue	–	–
		Gonophore	++ blue	++ magenta	–	++ magenta	–	<+ blue	–
	Outer layer	Hydrorhiza	x	x	x	x	x	x	x
		Hydrocaulus	++ purple	++ magenta	–	+++ magenta	+++ alcian blue	–	+ brown
		Hydranth	++ purple	+++ magenta	–	+++ magenta	+++ alcian blue	–	+ brown
		Gonophore	++ purple	+++ magenta	–	+ magenta	++ alcian blue	–	+ brown
*Pachycordyle michaeli*	Inner layer	Hydrorhiza	++ blue	+ magenta	–	+++ magenta	+ alcian blue	++ blue	+ yellow
		Hydrocaulus	++ blue	+ magenta	–	+++ magenta	–	++ blue	+ yellow
		Hydranth	ø	ø	ø	ø	ø	ø	ø
		Gonophore	+ blue	+ magenta	–	++ magenta	–	++ blue	<+ yellow
	Outer layer	Hydrorhiza	ø	ø	ø	ø	ø	ø	ø
		Hydrocaulus	ø	ø	ø	ø	ø	ø	ø
		Hydranth	ø	ø	ø	ø	ø	ø	ø
		Gonophore	ø	ø	ø	ø	ø	ø	ø
*Parawrightia robusta*	Inner layer	Hydrorhiza	++ blue	++ magenta	–	++ magenta	–	+++ blue	++ yellow
		Hydrocaulus	++ blue	++ magenta	–	++ magenta	–	++ blue	++ yellow
		Hydranth	+ blue	++ magenta	–	++ magenta	–	+ blue	+ yellow
	Outer layer	Hydrorhiza	+++ purple	+++ magenta	<+ magenta	+++ magenta	+++ alcian blue	–	–
		Hydrocaulus	+++ purple	+++ magenta	<+ magenta	+++ magenta	+++ alcian blue	–	–
		Hydranth	+++ purple	+++ magenta	–	+++ magenta	+++ alcian blue	–	–
*Rhizorhagium* sp.	Inner layer	Hydrorhiza	+++ blue	+++ magenta	<+ magenta	+++ magenta	–	+++ blue	+++ yellow
		Hydrocaulus	+++ blue	+++ magenta	<+ magenta	+++ magenta	–	+++ blue	+++ yellow
		Hydranth	+ blue	<+ magenta	–	+ magenta	<+ alcian blue	<+ blue	–
	Outer layer	Hydrorhiza	+++ purple	+ magenta	<+ magenta	++ magenta	+++ alcian blue	–	–
		Hydrocaulus	+++ purple	+ magenta	–	++ magenta	+++ alcian blue	–	–
		Hydranth	+++ purple	+ magenta	–	++ magenta	++ alcian blue	–	–
**Eudendriidae**									
*Eudendrium carneum*	Inner layer	Hydrorhiza	++ blue	++ magenta	–	++ magenta	–	+++ blue	+++ yellow
		Hydrocaulus	++ blue	++ magenta	–	++ magenta	–	+++ blue	+++ yellow
		Hydranth	ø	ø	ø	ø	ø	ø	ø
		Gonophore	+ blue	+ magenta	–	+ magenta	–	+ blue	<+ yellow
	Outer layer	Hydrorhiza	+++ blue	+ magenta	<+ magenta	+++ magenta	+++ alcian blue	–	–
		Hydrocaulus	+++ blue	+ magenta	<+ magenta	+++ magenta	+++ alcian blue	–	–
		Hydranth	ø	ø	ø	ø	ø	ø	ø
		Gonophore	ø	ø	ø	ø	ø	ø	ø
**Oceaniidae**									
*Turritopsis* sp.	Inner layer	Hydrorhiza	+++ blue	+++ magenta	–	+++ magenta	–	+++ blue	+++ yellow
		Hydrocaulus	+++ blue	+++ magenta	–	+++ magenta	–	+++ blue	+++ yellow
		Hydranth	+ blue	+ magenta	–	+ magenta	–	+ blue	?
	Membrane	Hydrorhiza	+++ purple	<+ magenta	–	++ magenta	++ alcian blue	–	–
		Hydrocaulus	+++ purple	<+ magenta	–	++ magenta	++ alcian blue	–	–
		Hydranth	+ purple	<+ magenta	–	++ magenta	+ alcian blue	–	–
**Pandeidae**									
*Leuckartiara* cf. *octona*	Inner layer	Hydrorhiza	+++ blue	+++ magenta	–	+++ magenta	–	+++ blue	++ yellow
		Hydrocaulus	+++ blue	+++ magenta	–	+++ magenta	–	+++ blue	++ yellow
		Hydranth	+ blue	+ magenta	–	+ magenta	–	++ blue	–
	Outer layer	Hydrorhiza	+++ purple	++ magenta	–	++ magenta	+++ alcian blue	–	–
		Hydrocaulus	+++ purple	++ magenta	–	++ magenta	+++ alcian blue	<+ blue	–
		Hydranth	+++ purple	++ magenta	–	++ magenta	+++ alcian blue	<+ blue	–
**Leptothecata**									
**Haleciidae**									
*Halecium bermudense*	Inner layer	Hydrorhiza	+++ blue	++ magenta	–	++ magenta	–	+++ blue	+ yellow
		Hydrocaulus	+++ blue	++ magenta	–	++ magenta	–	+++ blue	+ yellow
		Hydranth	+++ blue	++ magenta	–	++ magenta	–	+++ blue	+ yellow
		Gonophore	+++ blue	++ magenta	–	++ magenta	–	+++ blue	+ yellow
	Outer layer	Hydrorhiza	ø	ø	ø	ø	ø	ø	ø
		Hydrocaulus	ø	ø	ø	ø	ø	ø	ø
		Hydranth	ø	ø	ø	ø	ø	ø	ø
		Gonophore	ø	ø	ø	ø	ø	ø	ø
**Clytiidae**									
*Clytia gracilis*	Inner layer	Hydrorhiza	+++ blue	+++ magenta	–	+ magenta	++ alcian blue	+++ blue	+++ yellow
		Hydrocaulus	+++ blue	+++ magenta	–	+ magenta	++ alcian blue	+++ blue	+++ yellow
		Hydranth	++ blue	++ magenta	–	<+ magenta	++ alcian blue	++ blue	++ yellow
	Outer layer	Hydrorhiza	ø	ø	ø	ø	ø	ø	ø
		Hydrocaulus	ø	ø	ø	ø	ø	ø	ø
		Hydranth	ø	ø	ø	ø	ø	ø	ø
*Orthopyxis sargassicola*	Inner layer	Hydrorhiza	+++ blue	++ magenta	<+ magenta	++ magenta	–	++ blue	+yellow
		Hydrocaulus	++ blue	+ magenta	<+ magenta	++ magenta	–	++ blue	+ yellow
		Hydranth	++ blue	+ magenta	–	++ magenta	–	++ blue	+ yellow
		Gonophore	++ blue	+ magenta	<+ magenta	++ magenta	–	++ blue	+ yellow
	Outer layer	Hydrorhiza	ø	ø	ø	ø	ø	ø	ø
		Hydrocaulus	ø	ø	ø	ø	ø	ø	ø
		Hydranth	ø	ø	ø	ø	ø	ø	ø
		Gonophore	ø	ø	ø	ø	ø	ø	ø
**Obeliidae**									
*Obelia dichotoma*	Inner layer	Hydrorhiza	+++ blue	+++ magenta	<+ magenta	+++ magenta	–	+++ blue	++ yellow
		Hydrocaulus	+++ blue	+++ magenta	<+ magenta	+++ magenta	–	+++ blue	++ yellow
		Hydranth	+++ blue	++ magenta	–	+++ magenta	–	++ blue	++ yellow
		Gonophore	+++ blue	++ magenta	–	+++ magenta	–	+++ blue	++ yellow
	Outer layer	Hydrorhiza	ø	ø	ø	ø	ø	ø	ø
		Hydrocaulus	ø	ø	ø	ø	ø	ø	ø
		Hydranth	ø	ø	ø	ø	ø	ø	ø
		Gonophore	ø	ø	ø	ø	ø	ø	ø

**Notes.**

– not stained <+ nearly unstained + weakly stained ++ moderately stained +++ intensely stained x not analyzed histologically ø without structure * structure not identified ? doubtful reaction

**Figure 3:**
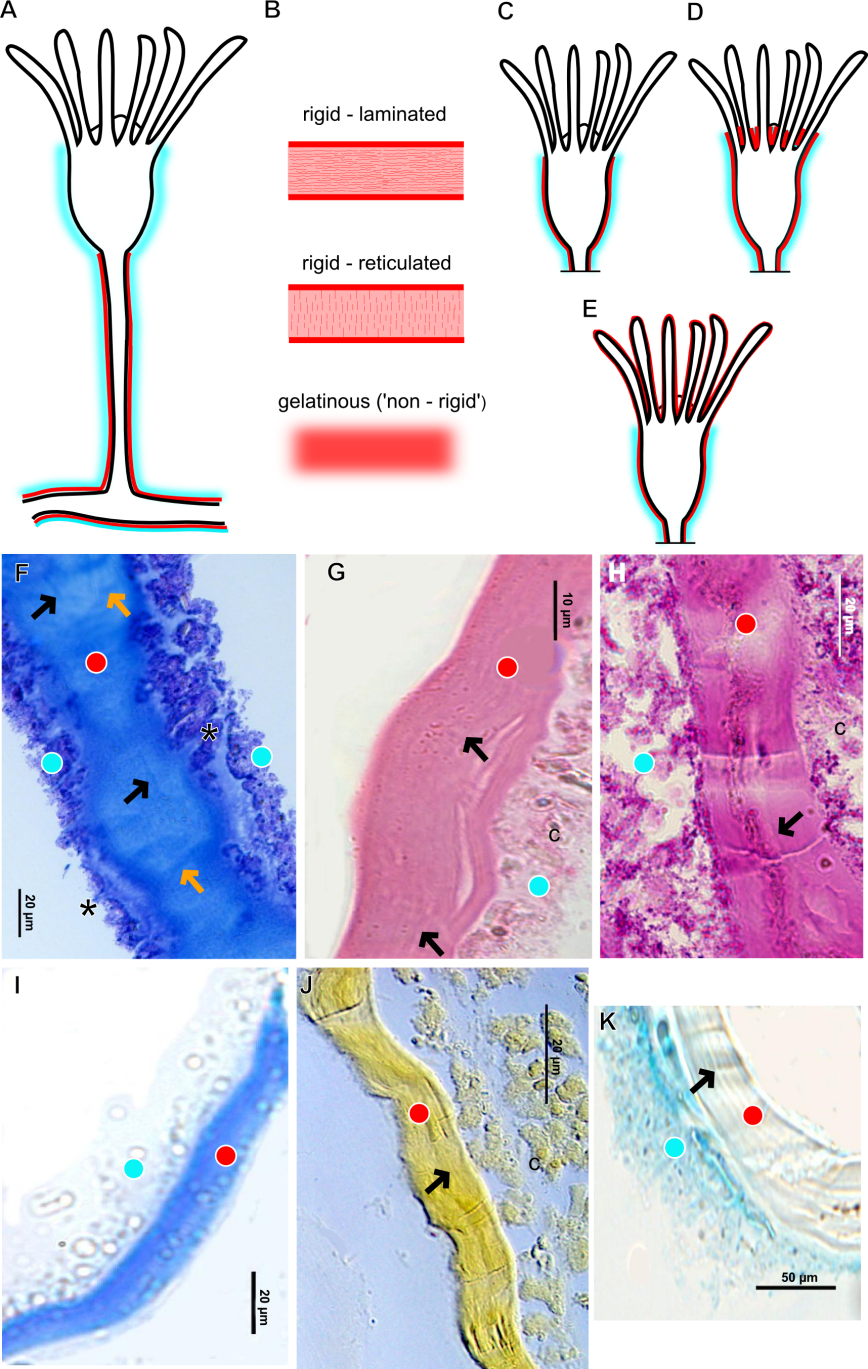
Exoskeletal structure of Bougainvilliidae Lütken, 1850. (A) Coverage of exoskeletal layers over hydrocaulus; (B) Histological structure of the inner layer (=perisarc) over hydrocaulus; (C)–(E) Coverage of exoskeletal layers over hydranth; (C) reaching the whorl of tentacles; (D) base of the tentacles; (E) inner layer entirely covering the tentacles; (F)–(K) Affinity for chemical tests and details of exoskeleton; (F) Toluidine blue; (G) Eosin; (H) Periodic acid-Schiff; (I) Mercury-bromophenol blue; (J) Naphthol yellow S; (K) Alcian blue pH 2.5. Cyan-blue line and circle indicate the outer layer of the exoskeleton (=exosarc), red line and circle indicate the inner layer of the exoskeleton, black arrow indicates laminae, orange arrow indicates transverse marks, asterisk indicates “perisarc extensions.” Abbreviation: c, coenosarc.

The exoskeleton of almost all species studied varies in thickness from region to region of the polyp as well as from species to species. The exoskeleton is divided into inner (=perisarc) and outer (=exosarc, as defined in Mendoza-Becerril et al., 2016) layers (Table 4), therefore corresponding to the bilayered exoskeleton (cf. Mendoza-Becerril et al., 2016) (Tables 5 and 7). The inner layer is continuous from the hydrorhiza to the base of the hydranth (Fig. 3A), rigid and laminated, sometimes reticulated or with a gelatinous (“non-rigid”) appearance (Fig. 3B). When the inner layer extends over the hydranth, reaching the base of the whorl of tentacles (Fig. 3C), the base of the tentacles (Fig. 3D) or even entirely enveloping the tentacles (Fig. 3E) it is thin and gelatinous. This layer has an affinity for TB (with a blue staining) (Fig. 3F), eosin (pink) (Fig. 3G), PAS (Fig. 3H), HgBpB (Fig. 3I) and NYS (Fig. 3J), and the intensity of the staining varies throughout the polyp (Tables 2 and 3), suggesting a chemical composition of aminopolysaccharides (AP) associated with proteins. The inner layer of some species has an affinity for AB (suggesting presence of GAGs) at the region of the hydrocaulus, in others this layer has no affinity for PAS (suggesting absence of AP).

The outer layer is usually thick and rugose, extending from the hydrorhiza to the hydranth, reaching the whorl of tentacles (Figs. 3A and 3C–3E) or covering up to the base of the tentacles (Fig. 3D). This layer has an affinity for TB (with a purple staining) (Fig. 3F), and is PAS- and AB-positive (Figs. 3H and 3K), suggesting a chemical composition of GAGs (Table 3). The outer layer is easily distinguished from the inner layer when treated with TB and AB techniques. However, it is difficult to distinguish both layers when the inner layer is thin or when the outer layer has no external material attached. The two layers may be connected by an anchoring system formed by extensions from the inner layer (“perisarc extensions”; Fig. 3F). The exosarc is frequently encrusted with thin organic and inorganic material (diatoms, mineral particles, bacterial film), therefore with a granular and rigid appearance (Figs. 3F–3I and 3K).

**Table:** 

**Superorder “Anthoathecata” Cornelius, 1992**
**Order Capitata Kühn, 1913** ***sensu stricto***
**Family Pennariidae McCrady, 1859**
***Pennaria disticha*** **Goldfuss, 1820**

**Figure 4:**
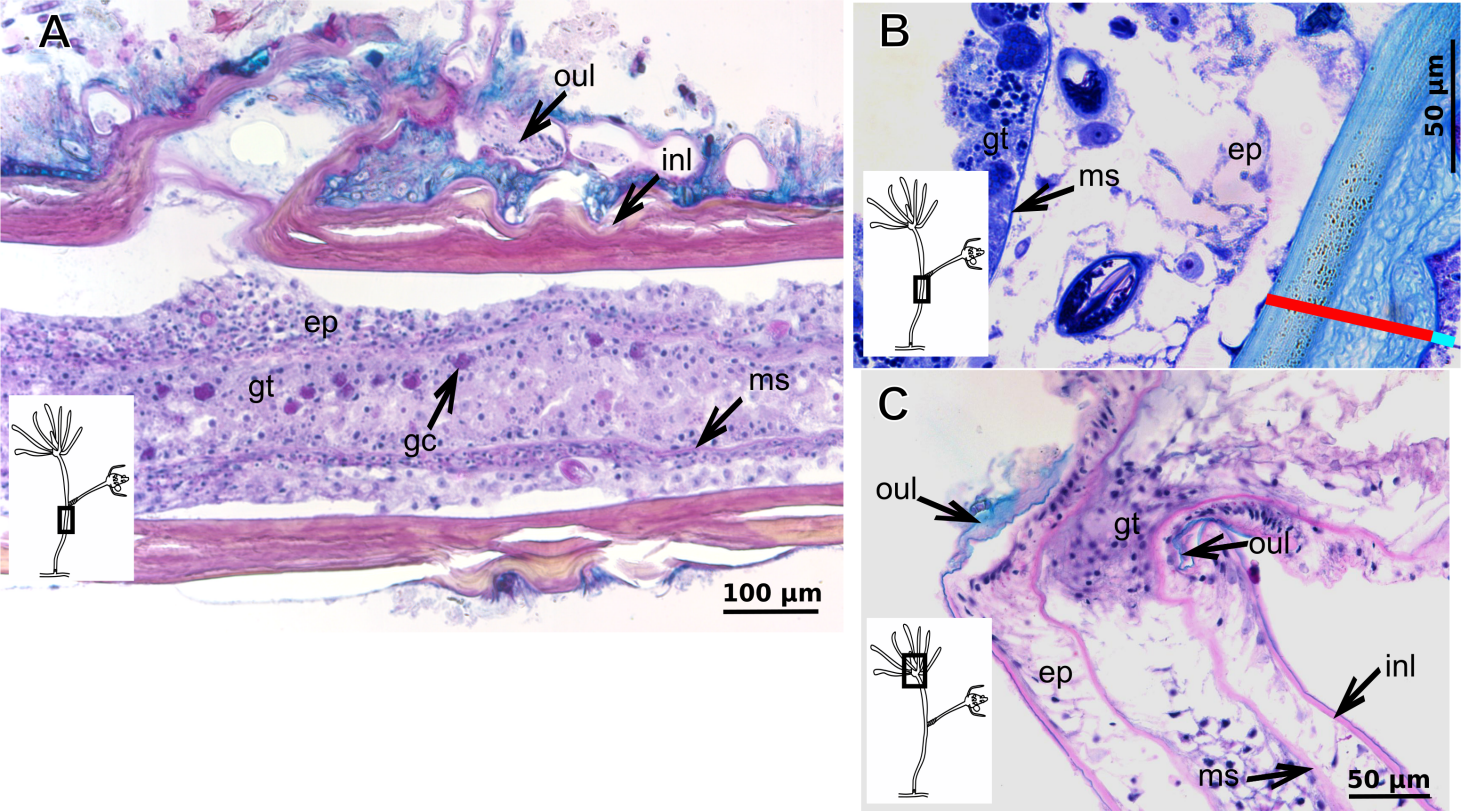
Internal and exoskeletal structure of *Pennaria disticha* Goldfuss, 1820. (A) Hydrocaulus and side-branch of the central region of the polyp, stained with AB + PAS + H; (B) exoskeleton of the central region of the polyp, stained with TB; (C) exoskeleton in the lower part of the hydranth, stained with AB + PAS + H. Cyan-blue line indicates the outer layer of the exoskeleton (=exosarc), red line indicates the inner layer of the exoskeleton (=perisarc). Abbreviations: ep, epidermis; gc, glandular cells; gt, gastrodermis; inl, inner layer; ms, mesoglea; oul, outer layer.

Bilayered semi-transparent or opaque exoskeleton. Inner layer laminated, fairly thick (Table 5), continuous from hydrorhiza to hydranth base then ending abruptly, annulated in basal regions (hydrocaulus and side-branches) and throughout hydrocaulus at more or less regular intervals (Figs. 4A–4C). Outer layer not rigid, thin (Table 5), continuous from hydrorhiza to hydranth base (Fig. 4C).

**Table:** 

**Order “Filifera” Kühn, 1913**
**Family Bougainvilliidae Lütken, 1850**
***Bimeria vestita*** **Wright, 1859**

**Figure 5:**
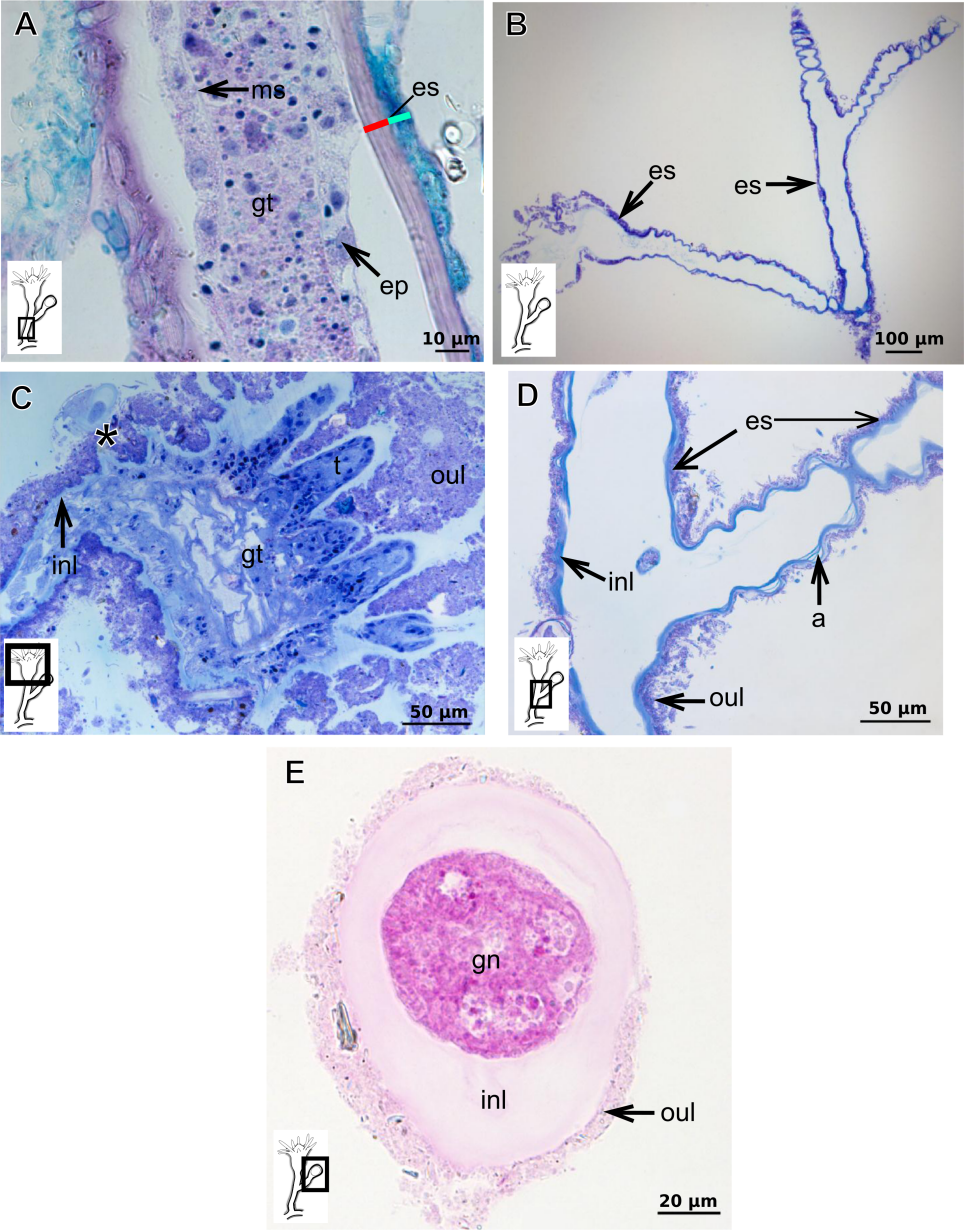
Exoskeletal structure of *Bimeria vestita* Wright, 1859. (A) Detail of hydrocauline exoskeleton, stained with AB + PAS + H; (B)–(D) stained with TB; (B) general organization of the exoskeleton of part of the colony; (C) exoskeleton of the hydranth; (D) detail of exoskeleton at side-branch and hydrocaulus; (E) detail of exoskeleton in female gonophore during development, stained with PAS. Cyan-blue line indicates the outer the layer of the exoskeleton (=exosarc), red line indicates the inner layer of the exoskeleton (=perisarc), asterisk indicates “perisarc extensions.” Abbreviations: a, annulation; ep, epidermis; es, exoskeleton; gn, gonadal cell cluster; gt, gastrodermis; inl, inner layer; ms, mesoglea; oul, outer layer; t, tentacle.

Bilayered exoskeleton. Inner layer rigid and laminated (Fig. 5A), moderately thick (Tables 5 and 7), continuous from hydrorhiza to tentacle base (Figs. 5B and 5C), frequently annulated on pedicels, or spirally corrugated at origin of side-branches (Figs. 5B–5D), also covering gonophore but in this case not rigid (Fig. 5E). Outer layer thick (Tables 5 and 7), rugose and encrusted with thin organic and inorganic material (diatoms, mineral particles), therefore appearing granular and rigid (Figs. 5A and 5D). Outer layer extends from hydrorhiza to tentacle base (Figs. 5A–5D), also on gonophore (Fig. 5E), becoming thinner (as a sheath) on tentacular bases and hypostome (Fig. 5C).

**Table 4:** Chemical and morphological types of exoskeleton and presence of anchoring structures.

Species/exoskeleton	Inner layer (=perisarc)			Outer layer (=exosarc)	Type of exoskeleton	Desmocytes	Perisarc extensions
	AP	Proteins	GAGs	GAGs			
**“Anthoathecata”**							
**Pennariidae**							
*Pennaria disticha*	+	+	x	+	Bilayered		x
**Bougainvilliidae**							
*Bimeria vestita*	+	+	x	+	Bilayered		+
*Bougainvillia muscus*	+	+	x	+	Bilayered		+
*Bougainvillia rugosa*	+	+	x	+	Bilayered		+
*Bougainvillia* sp.	+	+	x	+	Bilayered		+
*Dicoryne conferta*	+	+	+	+	Bilayered		+
*Garveia annulata*	+	+	+	+	Bilayered	Gonophore	+
*Garveia franciscana*	+	+	+	+	Bilayered		+
*Garveia gracilis*	+	+	x	+	Bilayered	Polyp	+
*Garveia nutans*	+	+	+	+	Bilayered	Polyp	+
*Pachycordyle michaeli*	+	+	x	ø	Corneous		x
*Parawrightia robusta*	+	+	x	+	Bilayered		+
*Rhizorhagium* sp.	+	+	x	+	Bilayered		x
**Eudendriidae**							
*Eudendrium carneum*	+	+	x	+	Bilayered		x
**Oceaniidae**							
*Turritopsis* sp.	+	+	x	ø	Corneous		x
**Pandeidae**							
*Leuckartiara* cf. *octona*	+	+	x	+	Bilayered	Polyp	+
**Leptothecata**							
**Haleciidae**							
*Halecium bermudense*	+	+	x	ø	Corneous	Hydrotheca	x
**Clytiidae**							
*Clytia gracilis*	+	+	+	ø	Corneous		x
*Orthopyxis sargassicola*	+	+	x	ø	Corneous		x
**Obeliidae**							
*Obelia dichotoma*	+	+	x	ø	Corneous		x

**Notes.**

AP aminopolysaccharides GAGs glycosaminoglycans + present x absent ø without layer

**Table 5:** Thickness of the exoskeleton (in µm) in Bougainvilliidae and other Hydroidolina.

Species	Inner layer (= perisarc)						Outer layer (=exosarc)				
	1	2	3	4	5	6	1	2	3	4	5
**“Anthoathecata”**											
**Pennariidae**											
*Pennaria disticha*	7.5	81.9–59.0	14.7	x	x	x	2.4	25.1–12.2	1.2	1.1	x
**Bougainvilliidae**											
*Bimeria vestita*	14.2	6.7	4.0	2.9	24.0	x	31.1	34.6	3.1	11.0	21.0
*Bougainvillia muscus*	25.2	20.5	4.6	1.4	x	x	5.4	3.1	8.4	1.8	x
*Bougainvillia rugosa*	11.1	14.7	x	1.6	2.9	x	x	132.5	15.0	5.0	3.9
*Bougainvillia* sp.	x	13.9	7.3	12.8	7.1	x	x	11.8	21.7	74.6	0.8
*Dicoryne conferta*	x	18.9	69.6	x	9.8	x	x	16.3	9.4	x	5.6
*Garveia annulata*	42.7	11.7	x	14.1	28.5	x	x	9.2	x	2.7	4.0
*Garveia franciscana*	x	24.9	14.9	3.6	6.0	x	x	17.0	11.8	9.9	6.2
*Garveia gracilis*	x	20.9	4.2	3.6	x	x	x	3.1	11.9	28.0	x
*Garveia nutans*	x	16.4	12.4	3.6	1.6	x	x	10.5	5.8	8.8	1.8
*Pachycordyle michaeli*	x	15.4–13.7	x	x	9.8	x	ø	ø	ø	ø	ø
*Parawrightia robusta*	x	7.0	x	5.1	x	x	x	96.6	x	15.4	x
*Rhizorhagium* sp.	18.5	14.9–10.3	x	2.1	x	2.1	18.6	8.5–1.4	x	2.7	x
**Eudendriidae**											
*Eudendrium carneum*	x	27.4–8.9	2.9	x	x	x	x	5.3–4.4	1.2	x	x
**Oceaniidae**											
*Turritopsis* sp.	5.4	9.1	x	1.4	x	x	ø	ø	ø	ø	ø
**Pandeidae**											
*Leuckartiara* cf. *octona*	9.6	9.2	x	2.8–1.6	x	1.1	29.4	16.8	x	67.5–9.0	x
**Leptothecata**											
**Haleciidae**											
*Halecium bermudense*	x	15.2–4.3	2.0	1.3	12.9	x	ø	ø	ø	ø	ø
**Clytiidae**											
*Clytia gracilis*	11.6	4.9	x	3.7	7.5	x	ø	ø	ø	ø	ø
*Orthopyxis sargassicola*	11.6	9.1	x	3.0	6.7	x	ø	ø	ø	øø	ø
**Family Obeliidae**											
*Obelia dichotoma*	9.7	9.7	x	2.9	2.5	x	ø	ø	ø	ø	ø

**Notes.**

1 hydrorriza 2 hydrocaulus 3 side-branch 4 hydranth 5 gonophore x not data ø without layer

**Table 6:** Synoptic table of exoskeletal characteristics in Bougainvilliidae.

Inner layer (=perisarc)									Outer layer (=exosarc)				
Species	Smooth	Laminated	Rigid	Reticulated	Annulations	Corrugated	Coverage	In gonophore	Rugose	Organic or inorganic material	Coverage	In gonophore	Figures
*Bimeria vestita*	–	*	*	–	On pedicels	Spirally in origin of side-branches	Hydrorhiza to tentacle base	p (not rigid)	*	p	Hydrorhiza to tentacle base	p (thinner as a sheath)	5
*Bougainvillia muscus*	*	*	–	*	–	x	Hydrorhiza to whorl of tentacles	x	*	p	Hydrorhiza to whorl of tentacle	x	6
*Bougainvillia rugosa*	*	*	–	*	–	x	Hydrorhiza to whorl of tentacles	p	*	p (rigid appearance)	Hydrorhiza to whorl of tentacles	p	6
*Bougainvillia* sp.	–	*	–	–	–	Irregularly	Hydrorhiza to whorl of tentacles	p (not laminated)	–	p	Hydrorhiza to whorl of tentacle	x	7
*Dicoryne conferta*	–	–	–	–	–	*	Hydrorhiza to lower part of hydranth	p	*	p	Hydrorhiza to lower part of hydranth	p	7
*Garveia annulata*	–	*	*	–	–	*	Hydrorhiza to whorl of tentacles	p (not rigid)	*	p	Hydrorhiza to whorl of tentacles	x	8
*Garveia franciscana*	–	*	–	*	On gonophore pedicels and origin of side-branches	Irregularly	Hydrorhiza to whorl of tentacles	p (not rigid)	*	p	Hydrorhiza to whorl of tentacles	p	9
*Garveia gracilis*	–	*	–	*	Origin of side-branches	–	Hydrorhiza to whorl of tentacles, not rigid at hydranth	x	*	p	Hydrorhiza to whorl of tentacles	x	10
*Garveia nutans*	–	*	*	–	–	Irregularly	Hydrorhiza to whorl of tentacles, and hydranth (not rigid)	p (not rigid)	*	p (rigid appearance)	Hydrorhiza to whorl of tentacles	p	10
*Pachycordyle michaeli*	–	*	–	–	–	*	Hydrorhiza to base hydranth	p (laminate)	ø	ø	ø	ø	11
*Parawrightia robusta*	–	*	–	*	–	Irregularly	Hydrorhiza to whorl of tentacles, not rigid in hydranth	x	*	p (rigid appearance)	Hydrorhiza to whorl of tentacles	x	12
*Rhizorhagium* sp.	–	*	–	–	–	Hydrocaulus	Hydrorhiza to tentacles	x	*	p (not rigid)	Hydrorhiza to hydranth	x	13

**Notes.**

p present x not data ø without layer - without feature * with feature

**Table 7:** Comparative measurements (µm) of some species of Bougainvilliidae collected in diferent locations.

Specimens and museum voucher	Region	Exoskeleton	
		Inner layer (= perisarc)	Outer layer (= exosarc)
***Bimeria vestita*** LEM-IBUSP_1	hydrorhiza	7.83	49.72
	hydrocaulus	4.63	69.52
	hydranth	4.12	^•^2.32
MZUSP5201	hydrorhiza	20.53	12.37
	hydrocaulus	8.00	19.00
	hydranth	2.52	19.82
UNMdP Hd3-38	hydrorhiza	x	x
	hydrocaulus	7.46	15.37
	hydranth	1.98	10.70
***Bougainvillia muscus*** LEM-IBUSP_2	hydrorhiza	2.20	3.59
	hydrocaulus	4.42	19.89
	hydranth	*	*
LEM-IBUSP_3	hydrorhiza	2.81	29.18
	hydrocaulus	1.61	10.40
	hydranth	x	x
MZUSP4217	hydrorhiza	x	x
	hydrocaulus	4.00	19.60
	hydranth	2.50	37.50
***Parawrightia robusta*** MZUSP 003390	hydrorhiza	*	*
	hydrocaulus	6.39	15.63
	hydranth	2.49	22.82
MZUSP 4379	hydrorhiza	*	*
	hydrocaulus	10.94	8.16
	hydranth	6.06	7.95

**Notes.**

x not analyzed • approximate measures, difficult define boundaries layer * it was not possible to define the boundaries of the layer

The measures of perisarc thickness were obtained from the position of maximum perisarc thickness.

***Bougainvillia muscus*** **(Allman, 1863)**

**Figure 6:**
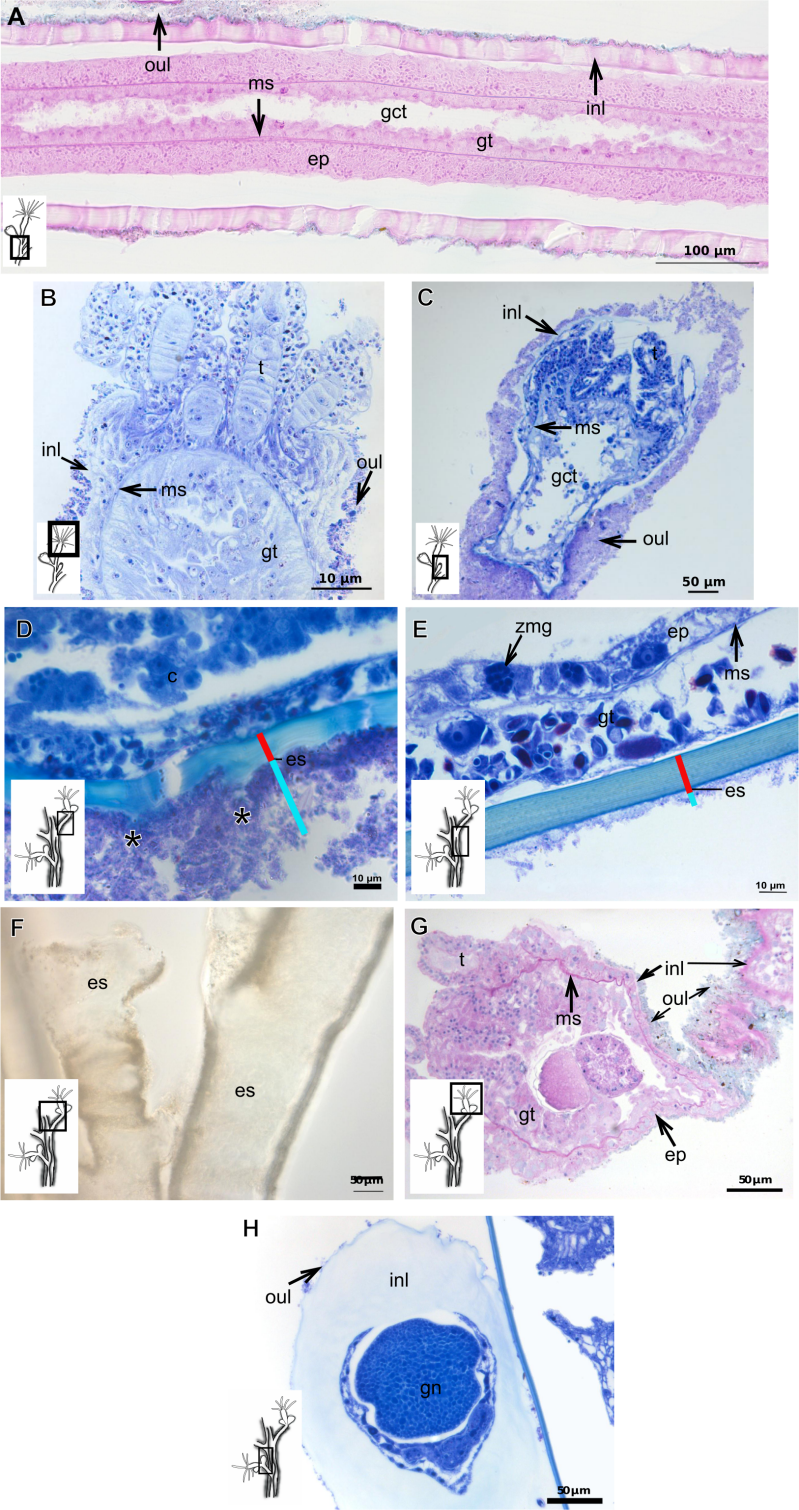
Exoskeletal structure. (A)–(C) *Bougainvillia muscus* (Allman, 1863); (D) general internal and external structure of the hydrocaulus, stained with AB + PAS + H; (E)–(F) contracted hydranth, stained with TB. (D)–(H) *Bougainvillia rugosa* Clarke, 1882. (D)–(E) stained with TB; (A) exoskeleton of the hydrocaulus; E: exoskeleton of the side-branch; (F) general exoskeleton of the polyp; (G) exoskeleton of the hydranth, stained with AB + PAS + H; (H) exoskeleton of mature female gonophore, stained with TB. Cyan-blue line indicates the outer layer of the exoskeleton (=exosarc), red line indicates the inner layer of the exoskeleton (=perisarc), asterisk indicates “perisarc extensions.” Abbreviations: c, coenosarc; ep, epidermis; es, exoskeleton; gct, gastrovascular cavity; gn, gonadal cell cluster; gt, gastrodermis; inl, inner layer; ms, mesoglea; oul, outer layer; t, tentacle; zmg, zymogen glandular cell.

Bilayered exoskeleton. Inner layer smooth, laminated and reticulated, thick (Tables 5 and 7), continuous from hydrorhiza to whorl of tentacles (Figs. 6A–6C). Outer layer thin (Tables 5 and 7), undulated and encrusted with organic and inorganic material (diatoms, sand grains, mud), therefore with granular appearance (Figs. 6A and 6C). Outer layer extends from hydrorhiza to whorl of tentacles (Fig. 6E). Both layers may fully cover the tentacles in the contracted hydranth (Figs. 6B–6C).

***Bougainvillia rugosa*** **Clarke, 1882**

Bilayered exoskeleton. Inner layer smooth, laminated and reticulated, thick (Figs. 6D–6E and Table 5), irregularly corrugated at origin of side-branches and at base of hydranth (Fig. 6F), continuous from hydrorhiza to whorl of tentacles (Fig. 6G), also covering gonophores (Fig. 6H). Outer layer fairly thick (Table 5), undulating and encrusted with inorganic material (detritus), therefore granular (Figs. 6D and 6E). Outer layer extends from hydrorhiza to whorl of tentacles (Fig. 6G), also covering gonophore (Fig. 6H).

***Bougainvillia*** **sp.**

**Figure 7:**
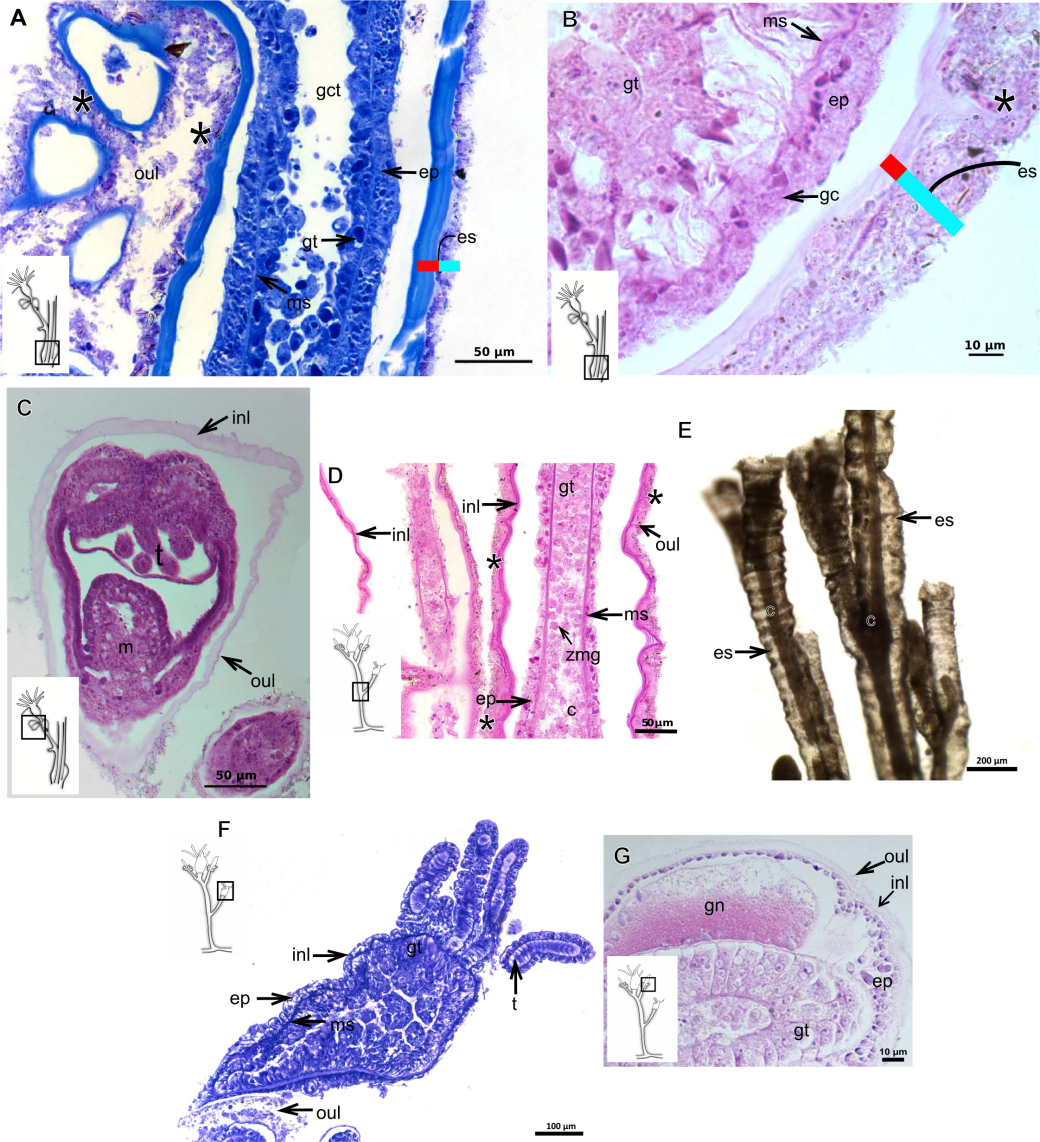
Exoskeletal structure. (A)–(C) *Bougainvillia* sp. (A) detail of hydrocauline exoskeleton, stained with TB; (B)–(C) stained with PAS; (B) transverse section of hydrocauline exoskeleton; (C) gonophore with complete medusa with manubrium and marginal tentacle linked to the bulb. (D)–(G) *Dicoryne conferta* Alder, 1856. (D) hydrocaulus of the central region of the polyp, stained with PAS; (E) external appearance of the exoskeleton; (F) exoskeleton of the hydranth, stained with TB; (G) mature female gonophore with sporosacs of styloid type, stained with HE. Cyan-blue line indicates the outer layer of the exoskeleton (=exosarc), red line indicates the inner layer of the exoskeleton (=perisarc), asterisk indicates “perisarc extensions.” Abbreviations: c, coenosarc; ep, epidermis; es, exoskeleton; gn, gonadal cell cluster; gt, gastrodermis; inl, inner layer; m, manubrium; ms, mesoglea; oul, outer layer; t, tentacle.

Bilayered exoskeleton. Inner layer laminated, irregularly corrugated, thick (Figs. 7A, 7B and Table 5); continuous from hydrorhiza to whorl of tentacles, not laminated at gonophore (Fig. 7C). Outer layer thick (Table 5), extending from hydrorhiza to whorl of tentacles and encrusted with detritus (Figs. 7A–7C).

***Dicoryne conferta*** **Alder, 1856**

Bilayered exoskeleton. Inner layer irregularly corrugated and thick (Table 5), continuous from hydrorhiza to lower part of hydranth (Figs. 7D–7F), also on gonophore (Fig. 7G), although not rigid. Outer layer thick (Table 5), wrinkled and with inorganic material (detritus). Outer layer extends from hydrorhiza to lower part of hydranth (Figs. 7D–7F), also on gonophore (Fig. 7G). Blastostyle without exoskeleton.

***Garveia annulata*** **Nutting, 1901**

**Figure 8:**
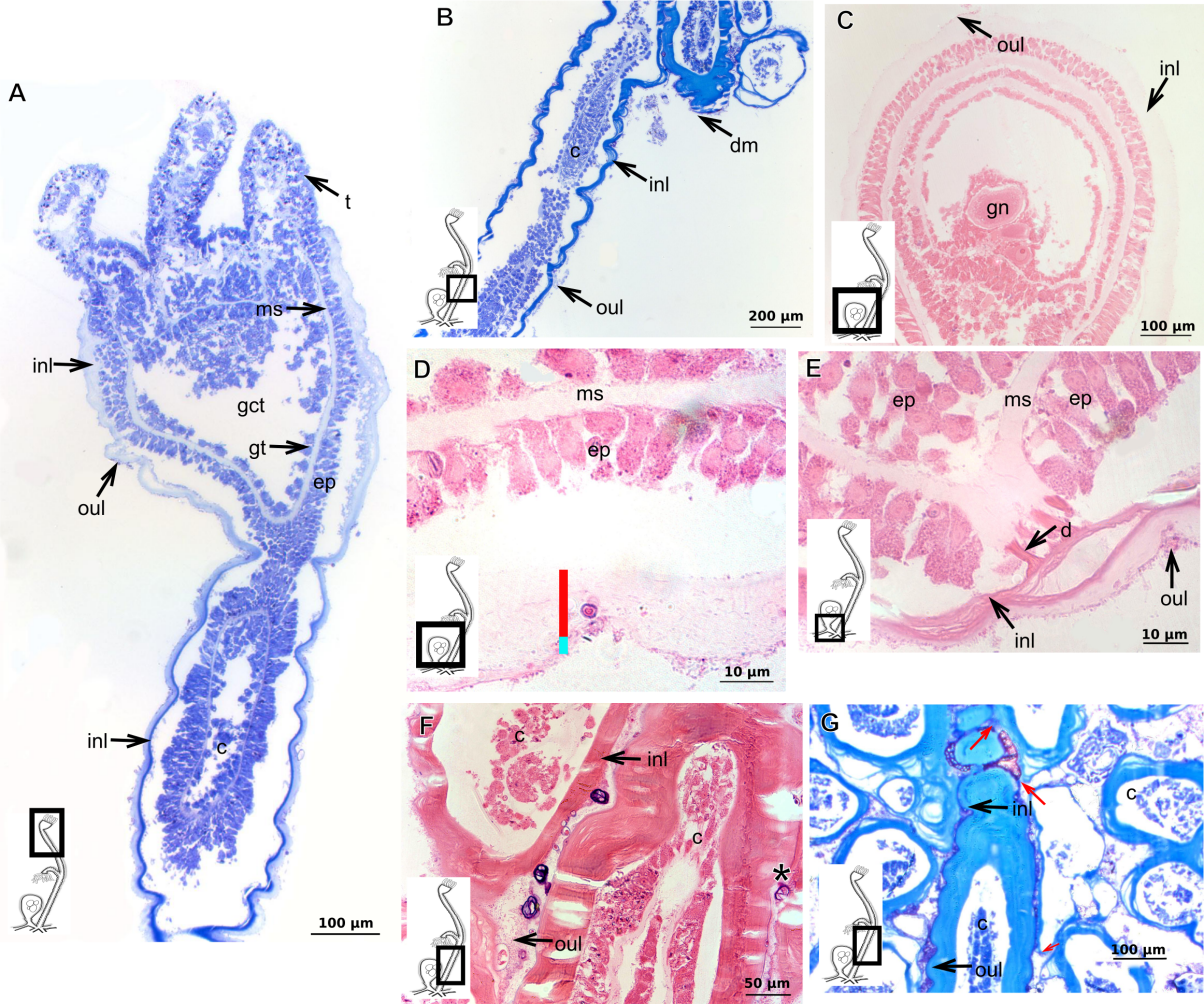
Exoskeletal structure of *Garveia annulata*  Nutting, 1901. (A)–(B) and (G) stained with TB; (A) hydrocauline and hydranth exoskeleton; (B) corrugated exoskeleton of the hydrocaulus; (C)–(F) stained with HE; (C) female gonophore showing sporosacs of the heteromedusoid type; (D) exoskeleton of female gonophore; (E) desmocytes in the female gonophore; (F)–(G) exoskeleton. Cyan-blue line indicates the outer layer of the exoskeleton (=exosarc), red line indicates the inner layer of the exoskeleton (=perisarc), asterisk indicates “perisarc extensions,” red arrow indicates the “exoskeletal connections” among the hydrocauline tubes. Abbreviations: c, coenosarc; d, desmocyte; dm, diatom; ep, epidermis; gct, gastrovascular cavity; gn, gonadal cell cluster; gt, gastrodermis; inl, inner layer; ms, mesoglea; oul, outer layer; t, tentacle.

Bilayered exoskeleton. Inner layer strongly laminated and rigid, corrugated, thick (Table 5), continuous from hydrorhiza to whorl of tentacles (Figs. 8A and 8B), also covering gonophore (Figs. 8C–8E) but in this case not rigid, except at the gonophore base. Outer layer moderately thick (Table 5), encrusted with organic (diatoms on base of hydrocaulus) and inorganic material (Figs. 8A and 8F–8G), therefore with granular appearance. Outer layer extends from hydrorhiza to whorl of tentacles (Fig. 8A), being discontinuous in hydrocaulus.

***Garveia franciscana*** **(Torrey, 1902)**

**Figure 9:**
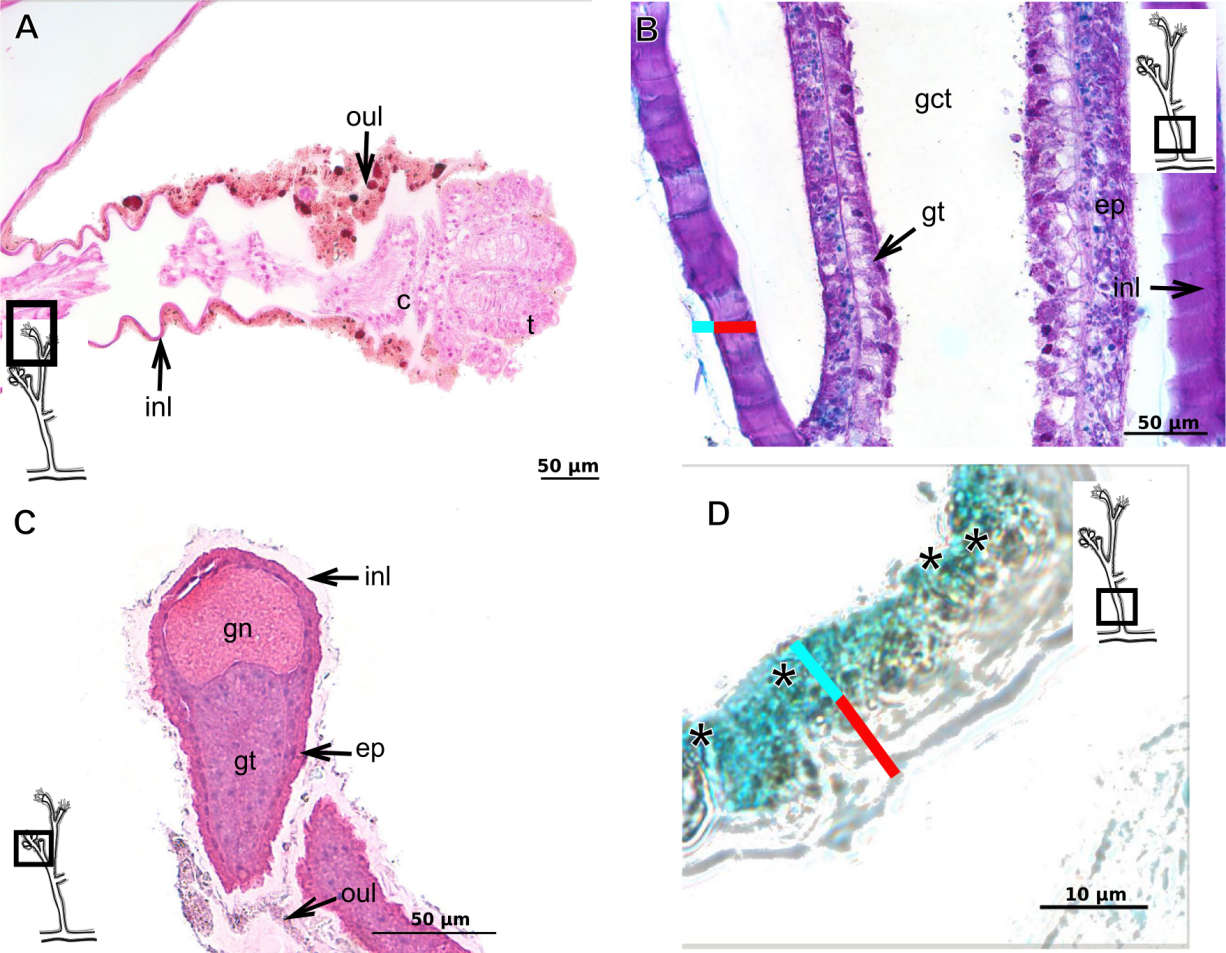
Exoskeletal structure of *Garveia franciscana* (Torrey, 1902). (A) Exoskeleton of the hydranth, stained with HE; (B) exoskeleton of the hydrocaulus, stained with AB+PAS+H; (C) exoskeleton of the female gonophore with sporosacs of the styloid type, stained with HE; (D) exoskeleton of the hydrocaulus, stained with AB. Cyan-blue line indicates the outer layer of the exoskeleton (=exosarc), red line indicates the inner layer of the exoskeleton (=perisarc), asterisk indicates “perisarc extensions.” Abbreviations: c, coenosarc; ep, epidermis; gct, gastrovascular cavity; gn, gonadal cell cluster; gt, gastrodermis; inl, inner layer; oul, outer layer; t, tentacle.

Bilayered exoskeleton. Inner layer laminated and reticulated, thick (Table 5), continuous from hydrorhiza to whorl of tentacles, irregularly corrugated, annulated on gonophore pedicels and at origins of side-branches (Figs. 9A and 8B), also covering gonophore but in this case not rigid (Fig. 9C). Outer layer granular, thick (Table 5), encrusted with organic and inorganic material (Fig. 9D), also covering gonophores (Fig. 9C).

***Garveia gracilis*** **(Clark, 1876)**

**Figure 10:**
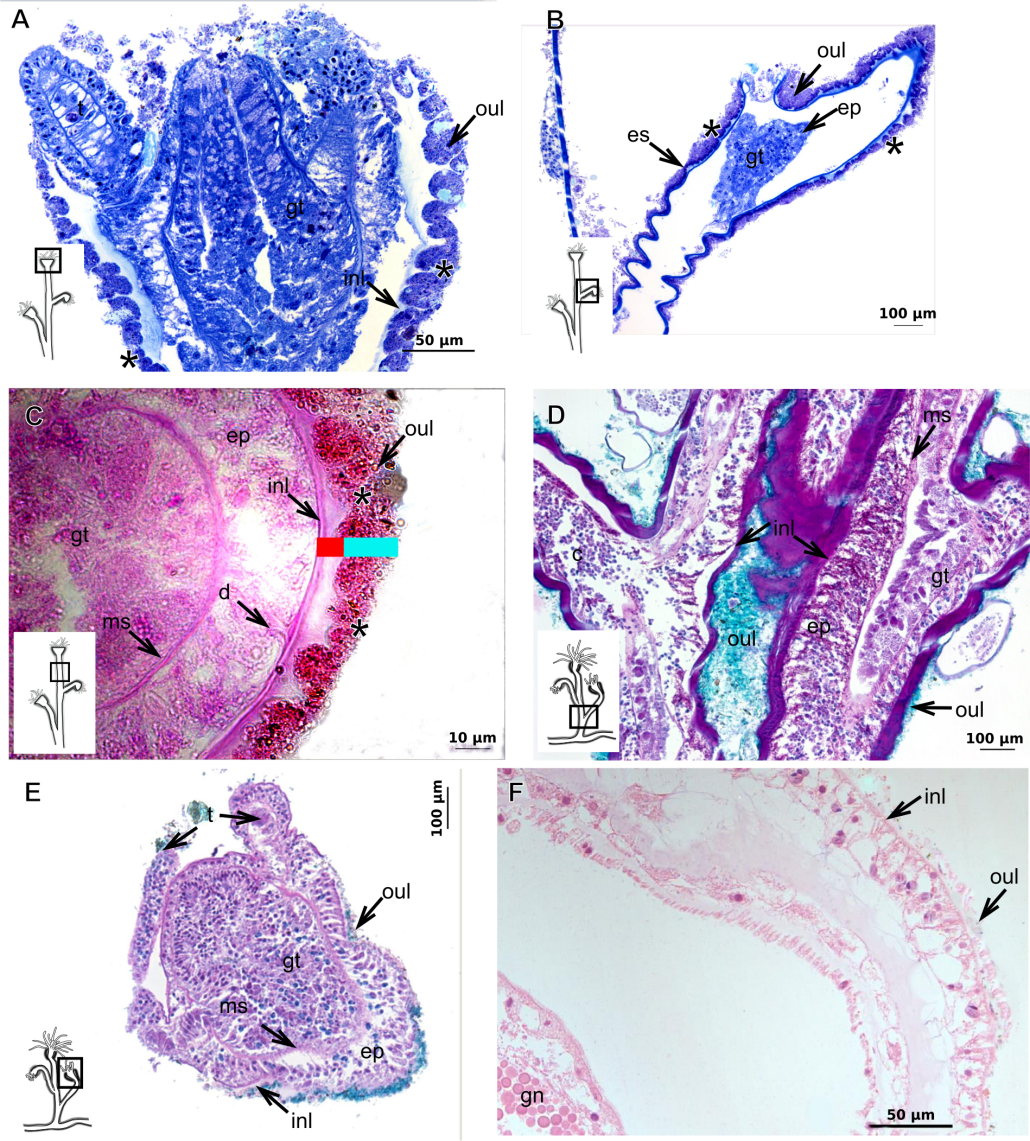
Exoskeletal structure. (A)–(C) *Garveia gracilis* (Clark, 1876). (A)–(B) stained with TB, (A) layers of the exoskeleton of the hydranth; (B) exoskeleton of the side-branch; (C) transverse section of the exoskeleton of the hydrocaulus, stained with PAS. (D)–(F) *Garveia nutans* Wright, 1859. (D)–(E) stained with AB + PAS + H; (D) exoskeleton of the hydrocaulus; (E) exoskeleton of the hydranth; (F) exoskeleton of the female gonophore, stained with HE. Cyan-blue line indicates the outer layer of the exoskeleton (=exosarc), red line indicates the inner layer of the exoskeleton (=perisarc), asterisk indicates “perisarc extensions.” Abbreviations: d, desmocyte; ep, epidermis; es, exoskeleton; gn, gonadal cell cluster; gt, gastrodermis; inl, inner layer; ms, mesoglea; oul, outer layer; t, tentacle.

Bilayered exoskeleton. Inner layer laminated and reticulated, thick (Table 5), continuous from hydrorhiza to whorl of tentacles (Fig. 10A), annulated at origin of side-branches (Fig. 10B), not rigid at hydranth. Outer layer thin (Table 5), densely encrusted with detritus (Figs. 10A–10C), continuous from hydrorhiza to whorl of tentacles, also may fully cover contracted hydranth and tentacles (Fig. 10A).

***Garveia nutans*** **Wright, 1859**

Bilayered exoskeleton. Inner layer laminated, irregularly corrugated and thick (Fig. 10D and Table 5), continuous from hydrorhiza to whorl of tentacles, also covering gonophore but in this case not rigid, also not rigid on hydranth (Figs. 10E and 10F). Outer layer thick (Table 5), continuous from hydrorhiza to whorl of tentacles (Fig. 10E), also covering gonophore (Fig. 10F), encrusted with detritus, therefore with rigid granular appearance (Fig. 10D).

***Pachycordyle michaeli*** **(Berrill, 1948)**

**Figure 11:**
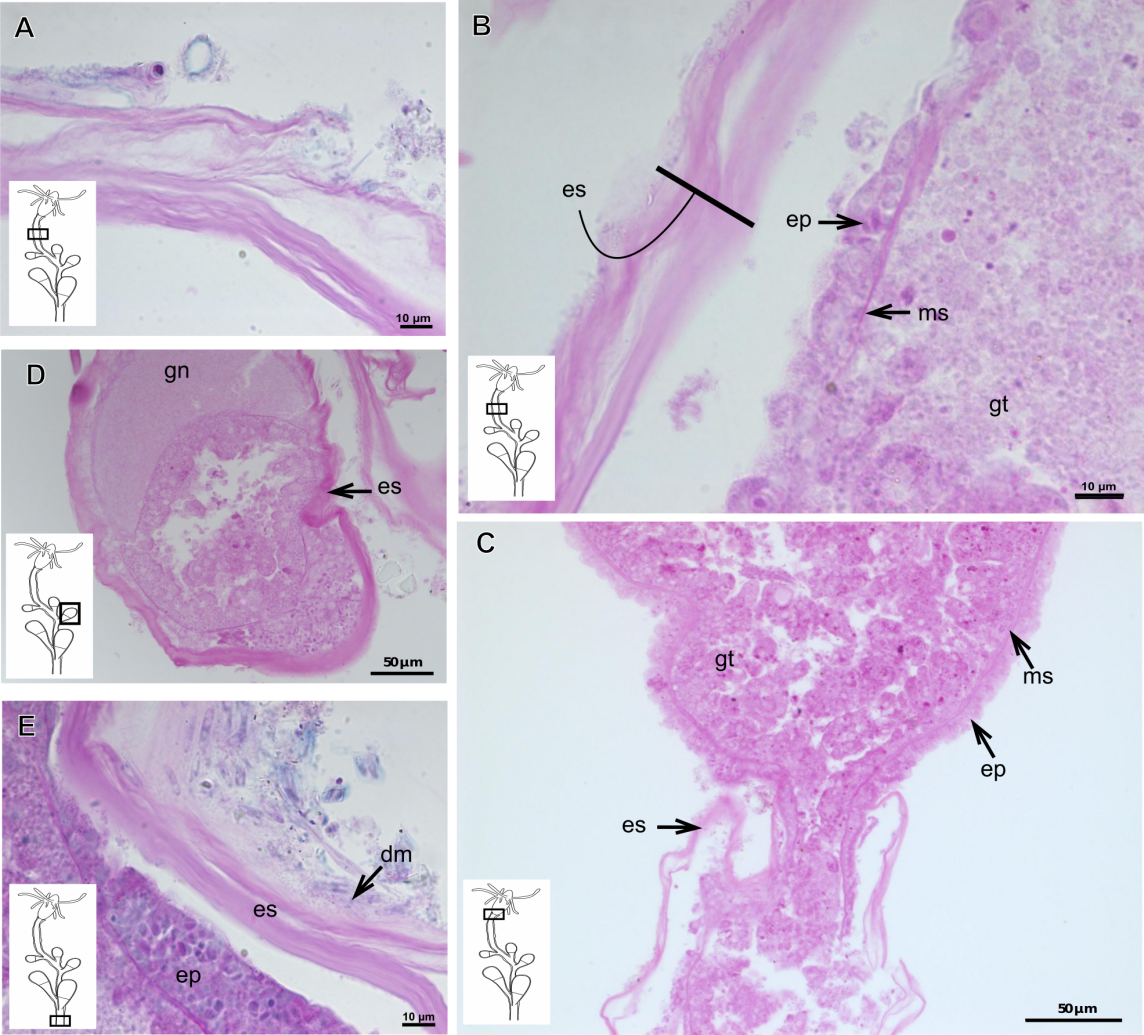
Exoskeletal structure of *Pachycordyle michaeli* (Berrill, 1948). (A) Hydrocauline exoskeleton, stained with AB + PAS + H; (B)–(D) stained with PAS; (B) hydrocauline exoskeleton; (C) exoskeleton at the base of the hydranth; (D) exoskeleton of the male gonophore with entocodon development; (E) exoskeleton with external material encrusted, stained with AB + PAS + H. Abbreviations: dm, diatoms; ep, epidermis; es, exoskeleton; gn, gonadal cell cluster; gt, gastrodermis; ms, mesoglea.

Corneous exoskeleton (chitin-protein) thick (Table 5), laminated with distinct series of sheets (Fig. 11A), corrugated, continuous from hydrorhiza to base of hydranth, irregularly corrugated at hydranth (Figs. 11B–11C), also covering gonophore where laminate is more consolidated (Fig. 11D). Exoskeleton encrusted with diatoms, particularly at hydrorhiza (Fig. 11E).

***Parawrightia robusta*** **Warren, 1907**

**Figure 12:**
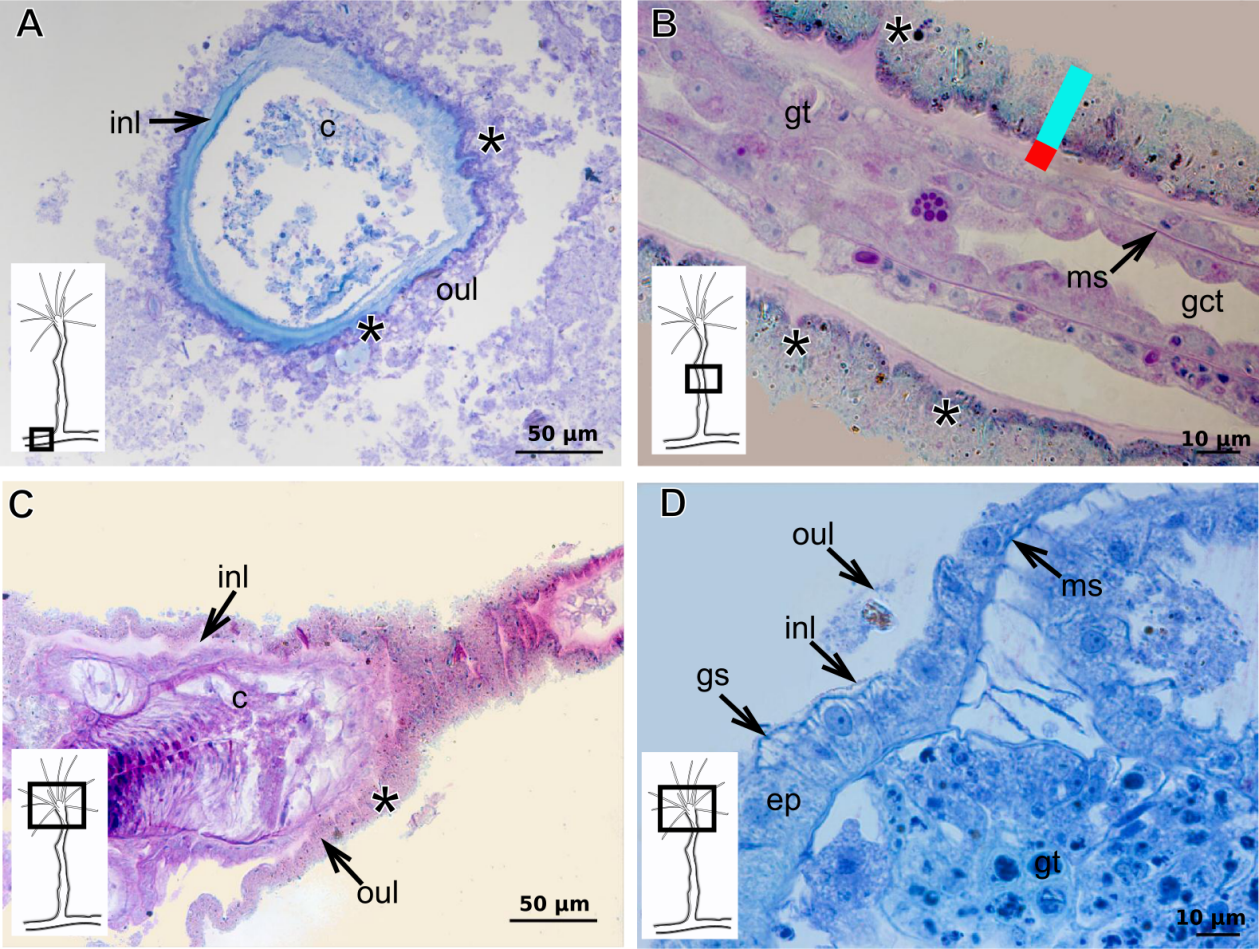
Exoskeletal structure of *Parawrightia robusta* Warren, 1907. (A) Transverse section of the exoskeleton of the hydrorhiza, stained with TB; (B)–(C) stained with AB + PAS + H; (B) hydrocauline exoskeleton; (C) exoskeleton of the hydranth; (D) exoskeleton at the tentacular base, stained with TB. Cyan-blue line indicates the outer layer of the exoskeleton (=exosarc), red line indicates the inner layer of the exoskeleton (=perisarc), asterisk indicates “perisarc extensions.” Abbreviations: c, coenosarc; ep, epidermis; gct, gastrovascular cavity; gs, secretory granules; gt, gastrodermis; inl, inner layer; ms, mesoglea; oul, outer layer.

Description: Bilayered exoskeleton. Inner layer laminated and reticulated, irregularly corrugated, moderately thick (Tables 5 and 7), continuous from hydrorhiza to whorl of tentacles (Figs. 12A–12D), not rigid on hydranth (Fig. 12C). Outer layer rugose and fairly thick (Tables 5 and 7), continuous from hydrorhiza to whorl of tentacles, encrusted with inorganic and organic material, therefore with rigid granular appearance (Figs. 12A–12D).

***Rhizorhagium*** **sp.**

**Figure 13:**
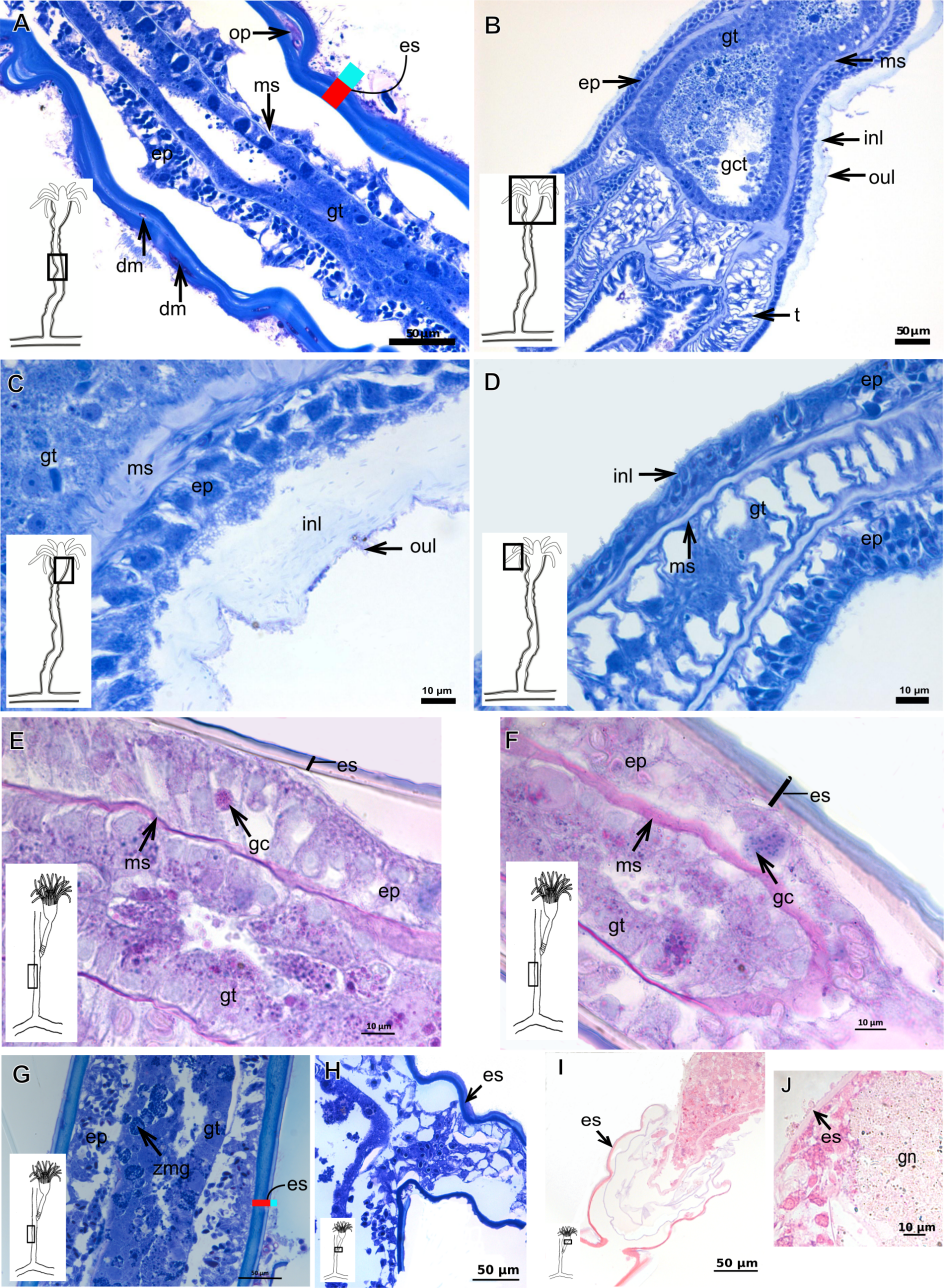
Exoskeletal structure. (A)–(D) *Rhizorhagium* sp. (A)–(D) stained with TB. (A) Hydrocaulus in the central region of the polyp; (B) hydranth; (C) exoskeleton of the hydranth; (D) exoskeleton of the tentacle. (E)–(J) *Eudendrium carneum* Clarke, 1882. (E)–(F) stained with AB + PAS + H; (E)–(G) Exoskeleton of the central region of the hydrocaulus; (G)–(H) stained with TB; (H) slightly corrugated exoskeleton of side-branch; (I) invagination of exoskeleton at the base of the hydranth, stained with HE; (J) exoskeleton in the gonophore, stained with PAS. Cyan-blue line indicates the outer layer of the exoskeleton (=exosarc), red line indicates the inner layer of the exoskeleton (=perisarc). Abbreviations: dm, diatoms; ep, epidermis; es, exoskeleton; gc, glandular cells; gct, gastrovascular cavity; gn, gonadal cell cluster; gt, gastrodermis; inl, inner layer; ms, mesoglea; op, organic particle; oul, outer layer; t, tentacle; zmg, zymogen glandular cell.

Bilayered exoskeleton. Inner layer rigid and laminated, occasionally corrugated at hydrocaulus (Fig. 13A), thick except at tentacles (Table 5), continuous from hydrorhiza to tentacles (Figs. 13B–13D). Inner layer with agglutinated organic particles (e.g., diatoms, Fig. 13A). Outer layer not rigid, generally thin (Table 5), except at hydrocaulus base covered with detritus and diatoms (Figs. 13A and 13C). Outer layer continuous from hydrorhiza to hydranth (Figs. 13A–13C).

**Table:** 

**Family Eudendriidae L. Agassiz, 1862**
***Eudendrium carneum*** **Clarke, 1882**

Bilayered exoskeleton, layers rigid, slightly corrugated at side-branch origin (Figs. 13E–13H), with invagination at hydranth base (Fig. 13I). Inner layer laminated and reticulated (Fig. 13G), moderately thick (Table 5), continuous from hydrorhiza to hydranth base, also covering gonophore (Fig. 13J). Outer layer homogeneous, moderately thick (Table 5), continuous from hydrorhiza to hydranth base (Figs. 13G and 13I) also covering gonophore (Fig. 13J). Outer layer stains intensely with AB, similar to outer layer of other bougainvilliids although with different coverage, i.e., not extending over hydranth. Hydrocaulus base encrusted with few organic and inorganic particles, similar to a third layer.

**Table:** 

**Family Oceaniidae Eschscholtz, 1829**
***Turritopsis*** **sp.**

**Figure 14:**
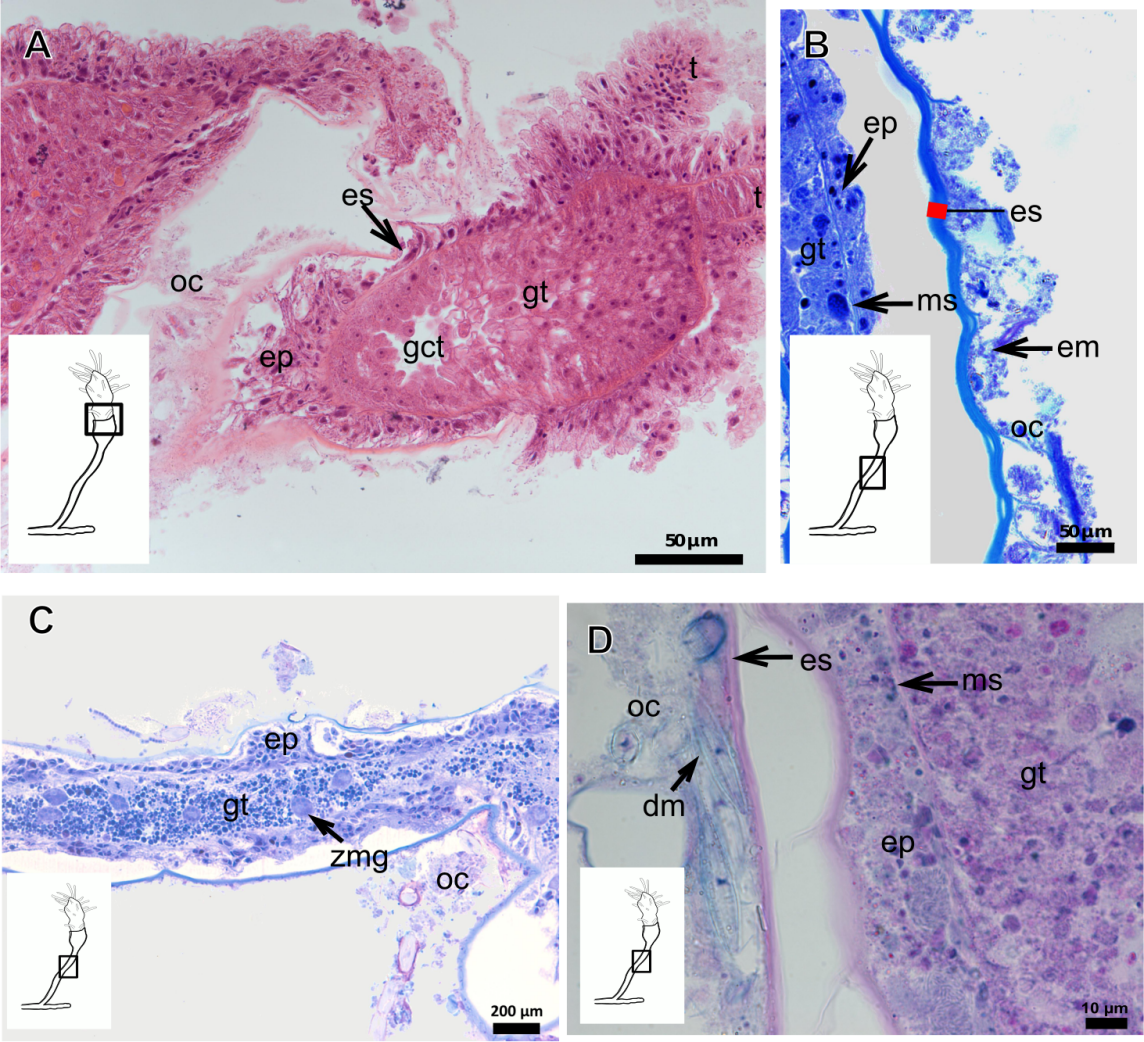
Internal and exoskeletal structure of *Turritopsis* sp. (A) Exoskeleton of the lower part of the hydranth, stained with HE; (B) irregularly corrugated exoskeleton of polyps, stained with TB; (C) granular outermost cover of the hydrocaulus, stained with TB; (D) exoskeleton with diatoms, stained with AB + PAS + H. Red line indicates the exoskeleton. Abbreviations: dm, diatoms; em, external material; ep, epidermis; es, exoskeleton; gct, gastrovascular cavity; gt, gastrodermis; ms, mesoglea; oc, outermost cover; t, tentacle; zmg, zymogen glandular cell.

Exoskeleton semi-transparent in developing polyps, pale cream-colored in developed polyps. Exoskeleton corneous (chitin-protein), moderately thick (Table 5), continuous from hydrorhiza to lower part of hydranth (below tentacles) (Figs. 14A–14B), occasionally corrugated in older polyps (Fig. 14B) and encrusted with organic and inorganic material under natural conditions, especially at hydrorhiza and hydrocaulus base. Exoskeleton with an outer covering (Figs. 14B–14D), which stains weakly with AB, suggesting low concentration of GAGs. Outer covering (Figs. 14A–14D) encrusted with abundant external material, with granular appearance, irregularly continuous from hydrorhiza to lower part of hydranth.

**Table:** 

**Family Pandeidae Haeckel, 1879**
***Leuckartiara* cf.*octona* (Fleming, 1823)**

**Figure 15:**
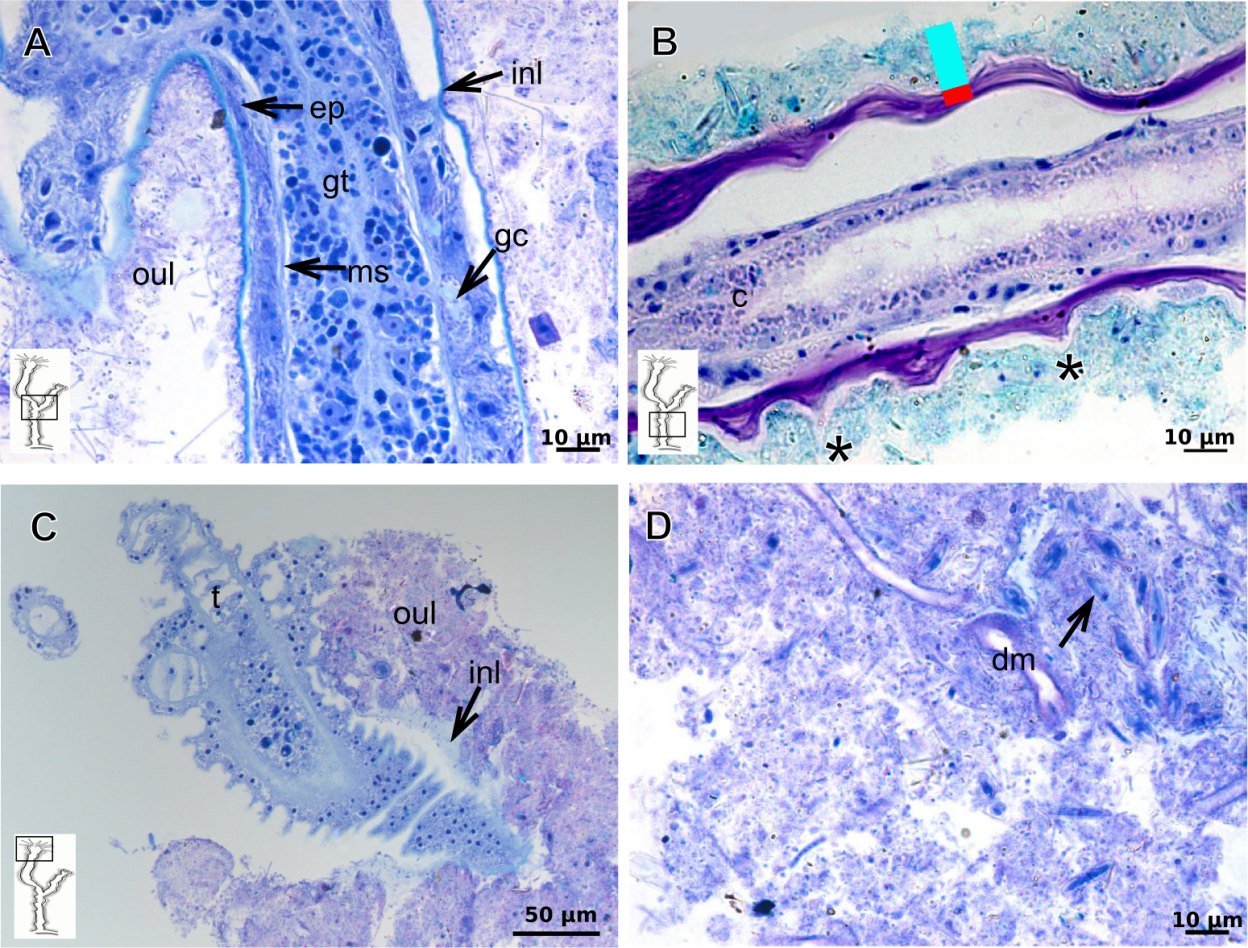
Internal and exoskeletal structure of *Leuckartiara* cf. *octona* (Fleming, 1823). (A) Hydrocaulus and side-branch of the central region of the polyp, stained with TB; (B) hydrocauline exoskeleton, stained with AB + PAS + H; (C)–(D) stained with TB; (C) exoskeleton of the hydranth; (D) outer layer (=exosarc) with organic and inorganic material. Cyan-blue line indicates the outer layer of the exoskeleton, red line indicates the inner layer of the exoskeleton (=perisarc), asterisk indicates “perisarc extensions.” Abbreviations: c, coenosarc; dm, diatoms; ep, epidermis; gc, glandular cells; gt, gastrodermis; inl, inner layer; ms, mesoglea; oul, outer layer; t, tentacle.

Bilayered exoskeleton. Inner layer rigid and laminated, moderately thick (Table 5), irregularly corrugated at hydrocaulus (Figs. 15A and 15B) and continuous from hydrorhiza to tentacles (Fig. 15C). Outer layer thick (Table 5), undulating (Fig. 15B), encrusted with organic (diatoms) and inorganic material (detritus) (Figs. 15B–15D). Outer layer extends from hydrorhiza to whorl of tentacles (Fig. 15C) and in free stolon/branch with single “non-rigid” layer.

### Exoskeleton organization in leptothecates

Hydroids of Clytiidae and Obeliidae with glandular cells, with affinity for TB, PAS, HgBpB and NYS (Table 3), abundant at hydrocaulus and gonophore base. I-cells rarely observed. Haleciid hydroids with vacuolated glandular cells with affinity for TB, PAS, and HgBpB, moreover with i-cells in the hydrocaulus.

**Figure 16:**
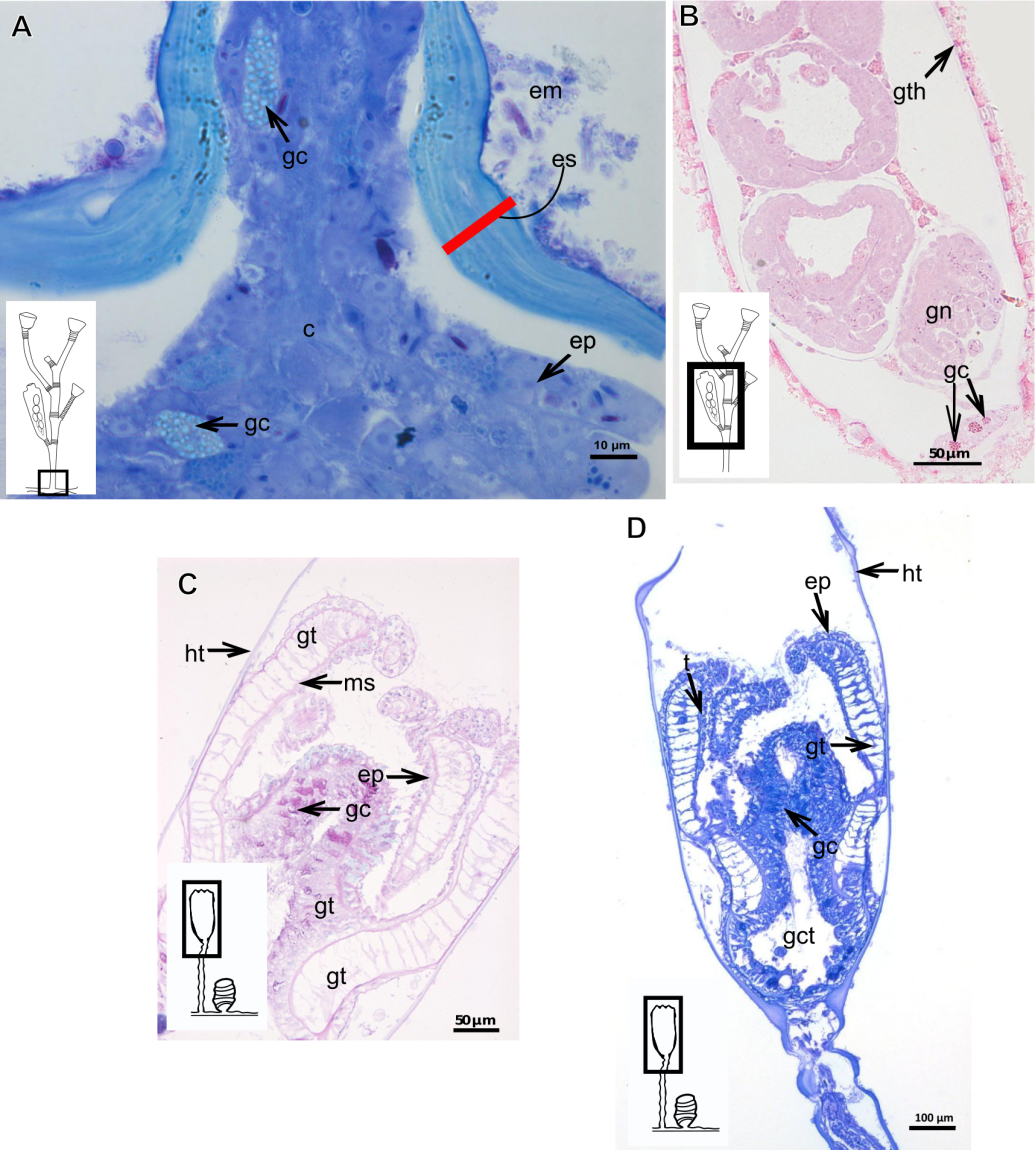
Coenosarc of species of Clytiidae Cockerell, 1911 and Obeliidae Maronna et al., 2016. (A)–(B) *Obelia dichotoma* (Linnaeus, 1758). (A) Hydrorhiza with glandular cells and exoskeleton, stained with TB; (B) exoskeleton of gonotheca and epidermal glandular cells, stained with HE; (C)–(D) *Orthopyxis sargassicola* (Nutting, 1915). (C) Hydranth, stained with AB + PAS + H; (D) hydranth, stained with TB. Red line indicates the exoskeleton. Abbreviations: c, coenosarc; em, external material; ep, epidermis; es, exoskeleton; gc, glandular cells; gct, gastrovascular cavity; gt, gastrodermis; gth, gonotheca; ht, hydrotheca; ms, mesoglea; t, tentacle.

The exoskeleton is corneous (chitin-protein) with different thickness (Tables 4 and 5), laminated, covering different regions of polyp (Figs. 16A–16D), sometimes with associated organic (diatoms) and inorganic material (Fig. 16A). Exoskeleton with affinities for TB, PAS, HgBpB and NYS in some species, but with weak affinity for AB (Tables 2 and 3). Association with diatoms may depend on environmental conditions.

**Table:** 

**Superorder Leptothecata Cornelius, 1992**
**Order Macrocolonia Leclère et al., 2009**
**Suborder Haleciida Bouillon, 1984** ***sensu*** **Maronna et al., 2016**
**Haleciidae Hincks, 1868**
***Halecium bermudense*** **Congdon, 1907**

**Figure 17:**
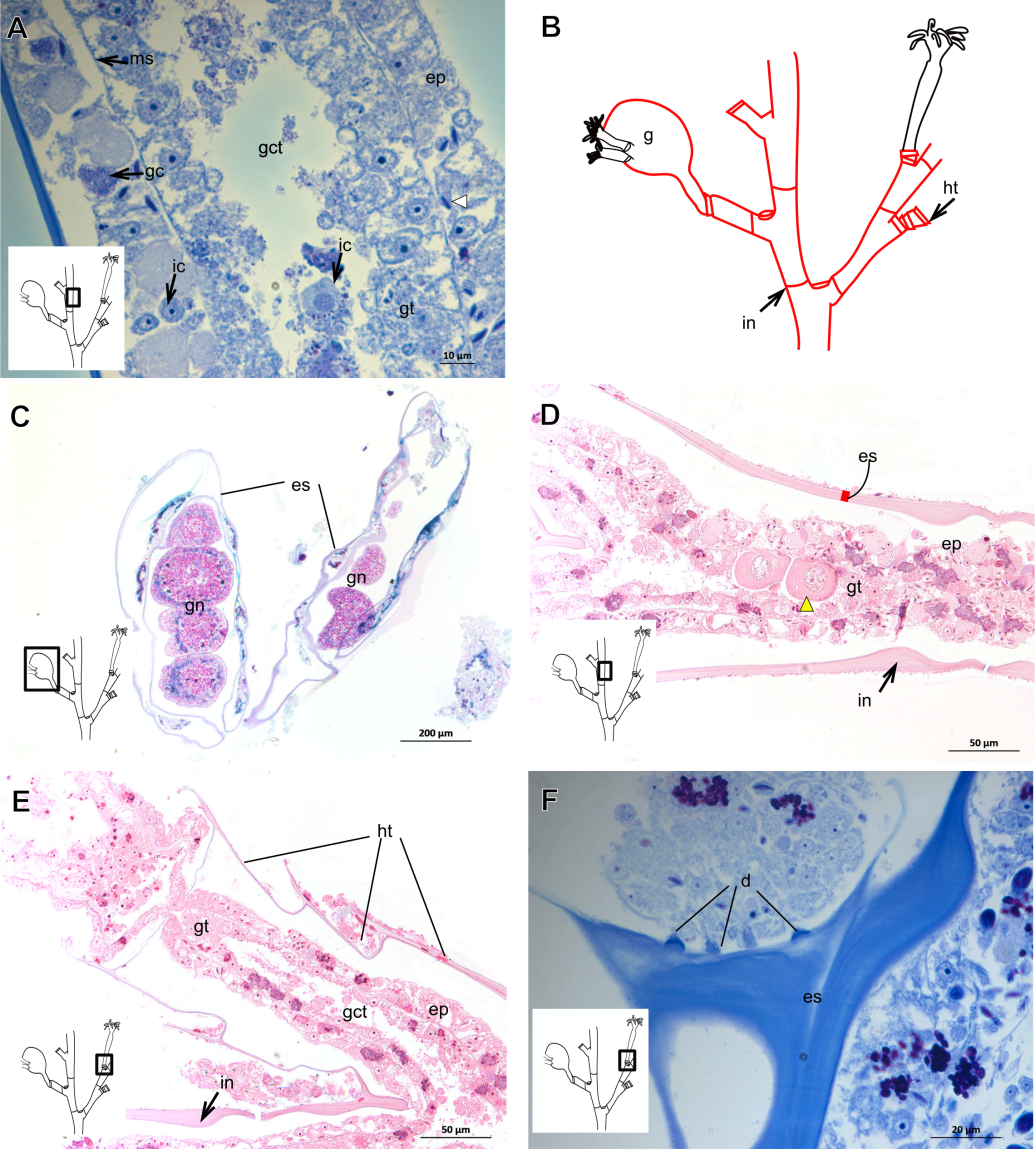
Coenosarc and exoskeletal structure of *Halecium bermudense* Congdon, 1907. (A) Hydrocauline coenosarc, stained with TB; (B) general morphology of the polyp; (C) exoskeleton of the female gonophore, stained with AB + PAS + H; (D)–(E) stained with HE; (D) hydrocauline exoskeleton; (E) primary and secondary hydrothecae; (F) primary hydrothecae, stained with TB. Yellow arrowhead indicates the zooxanthellae, red line indicates the exoskeleton (=perisarc). Abbreviations: d, desmocytes; ep, epidermis; es, exoskeleton; g, gonophore; gc, glandular cells; gct, gastrovascular cavity; gn, gonadal cell cluster; gt, gastrodermis; ht, hydrotheca; ic, interstitial cells; in, internode; ms, mesoglea.

Exoskeleton corneous (chitin-protein), rigid but not laminated, thin (Fig. 17A and Table 5), continuous from hydrorhiza to lower part of hydranth (Fig. 17B), also on gonophores (=gonotheca) (Figs. 17B and 17C). Exoskeleton forming internodes throughout polyp, primary hydrotheca, secondary hydrotheca and pedicel of secondary hydrotheca at hydranth base and lower part of hydranth (Figs. 17D–17F). Hydrotheca with desmocytes (Fig. 17F) and i-cells at the base of the hydrocaulus epidermis.

**Table:** 

**Order Statocysta Leclère et al., 2009**
**Suborder Proboscoida Broch, 1910** ***sensu*** **Maronna et al., 2016 and Cunha, Collins & Marques, 2017**
**Infraorder Obeliida** **Maronna et al., 2016**
**Family Clytiidae Cockerell, 1911**
***Clytia gracilis*** **(M. Sars, 1850)**

**Figure 18:**
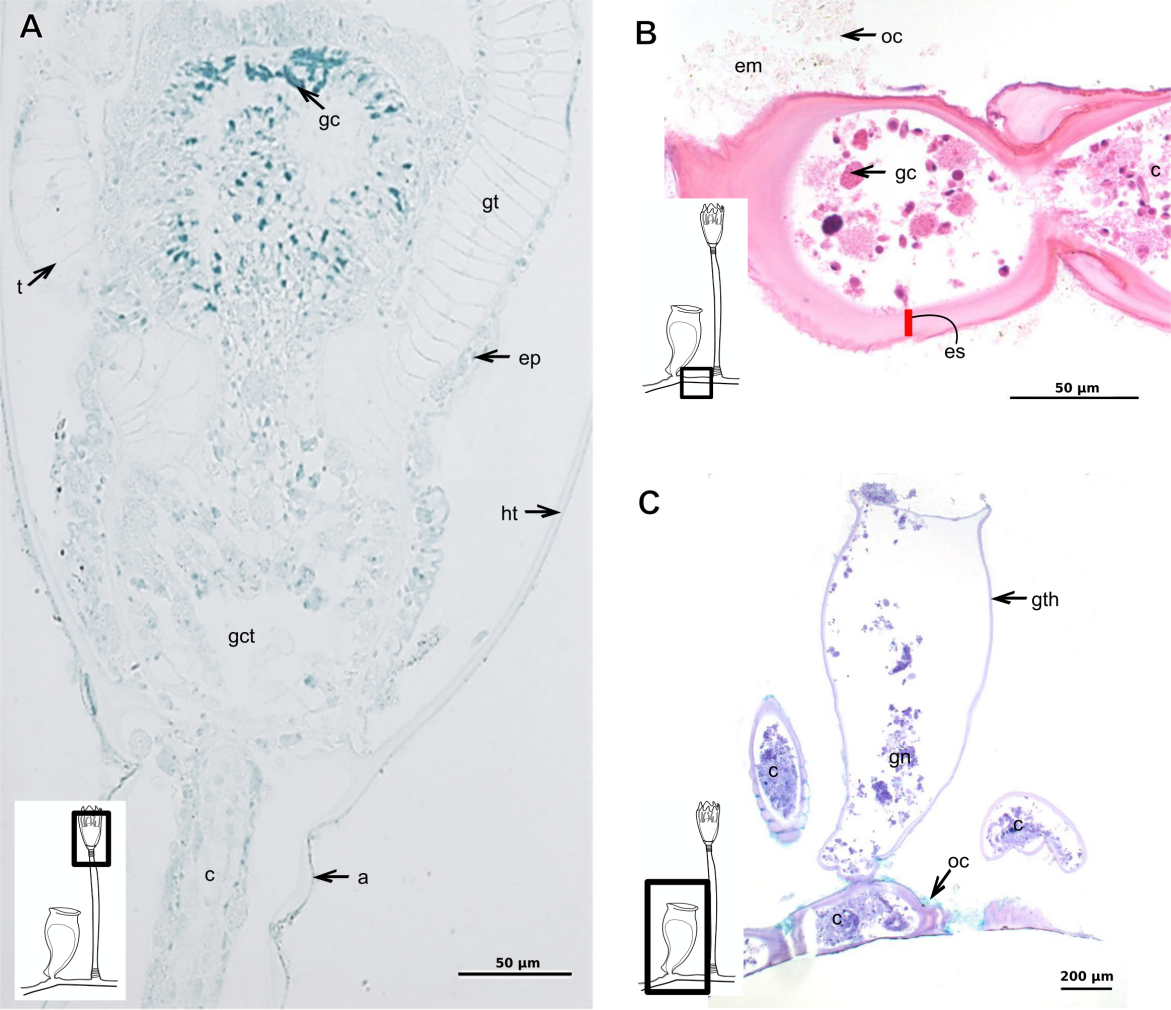
Exoskeletal structure in specimens of *Clytia gracilis* (M. Sars, 1850). (A) Exoskeleton of the hydranth, stained with HgBpB; (B) exoskeleton of the hydrorhiza, stained with HE; (C) exoskeleton of gonotheca, stained with AB + PAS + H. Red line indicates the layer of exoskeleton (=perisarc). Abbreviations: a, annulation; c, coenosarc; em, external material; ep, epidermis; es, exoskeleton; gc, glandular cells; gct, gastrovascular cavity; gt, gastrodermis; gth, gonotheca; ht, hydrotheca; oc, outermost cover; t, tentacle.

Exoskeleton corneous (chitin-protein), semi-transparent, laminated, smooth, thick (Table 5), continuous from hydrorhiza to hydranth, annulated at distal and proximal regions of hydrocaulus (Figs. 18A–18C). External region (in contact with environment) stains more intensely with AB than internal part (in contact with coenosarc), showing larger amount of GAGs. Some regions with an outer covering formed by substances produced either by diatoms associated with exoskeleton or by natural substrates (e.g., mollusks) (“external material” in Figs. 18B and 18C).

**Figure 19:**
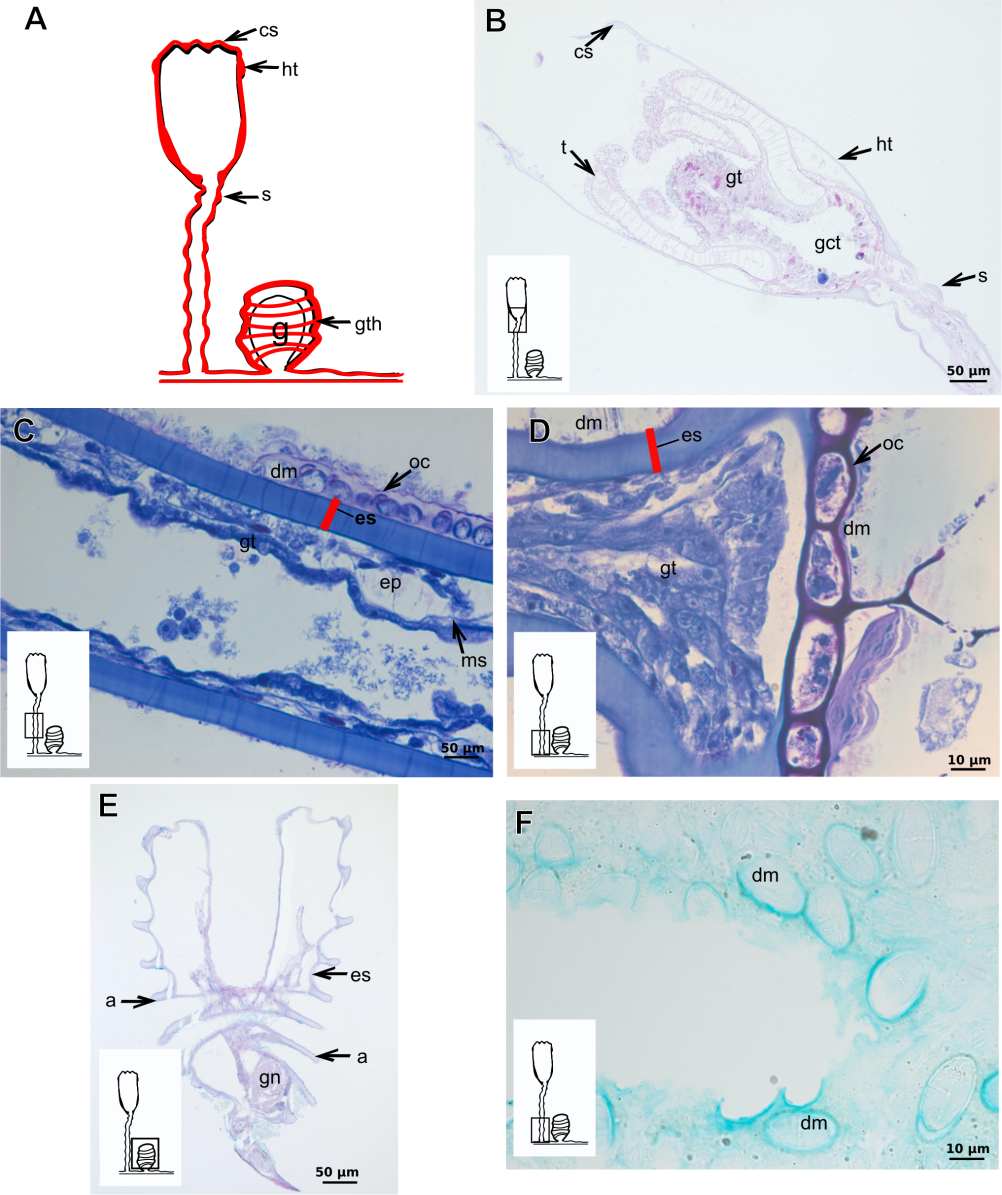
Exoskeletal structure of *Orthopyxis sargassicola* (Nutting, 1915). (A) General morphology of the polyp; (B) exoskeleton of the hydrotheca and subhydrothecal spherule, stained with AB + PAS + H; (C)–(D) stained with TB; (C) hydrocaulus; (D) exoskeleton with diatoms at the base of the hydrocaulus; (E) gonotheca, stained with AB + PAS + H; (F) diatoms on the exoskeleton of the hydrocaulus, stained with AB. Red line indicates the exoskeleton (=perisarc). Abbreviations: a, annulation; cs, cusps; dm, diatoms; ep, epidermis; es, exoskeleton; g, gonophore; gct, gastrovascular cavity; gn, gonadal cell cluster; gt, gastrodermis; gth, gonotheca; ht, hydrotheca; ms, mesoglea; s, spherule; oc, outermost cover; t, tentacle.

**Figure 20:**
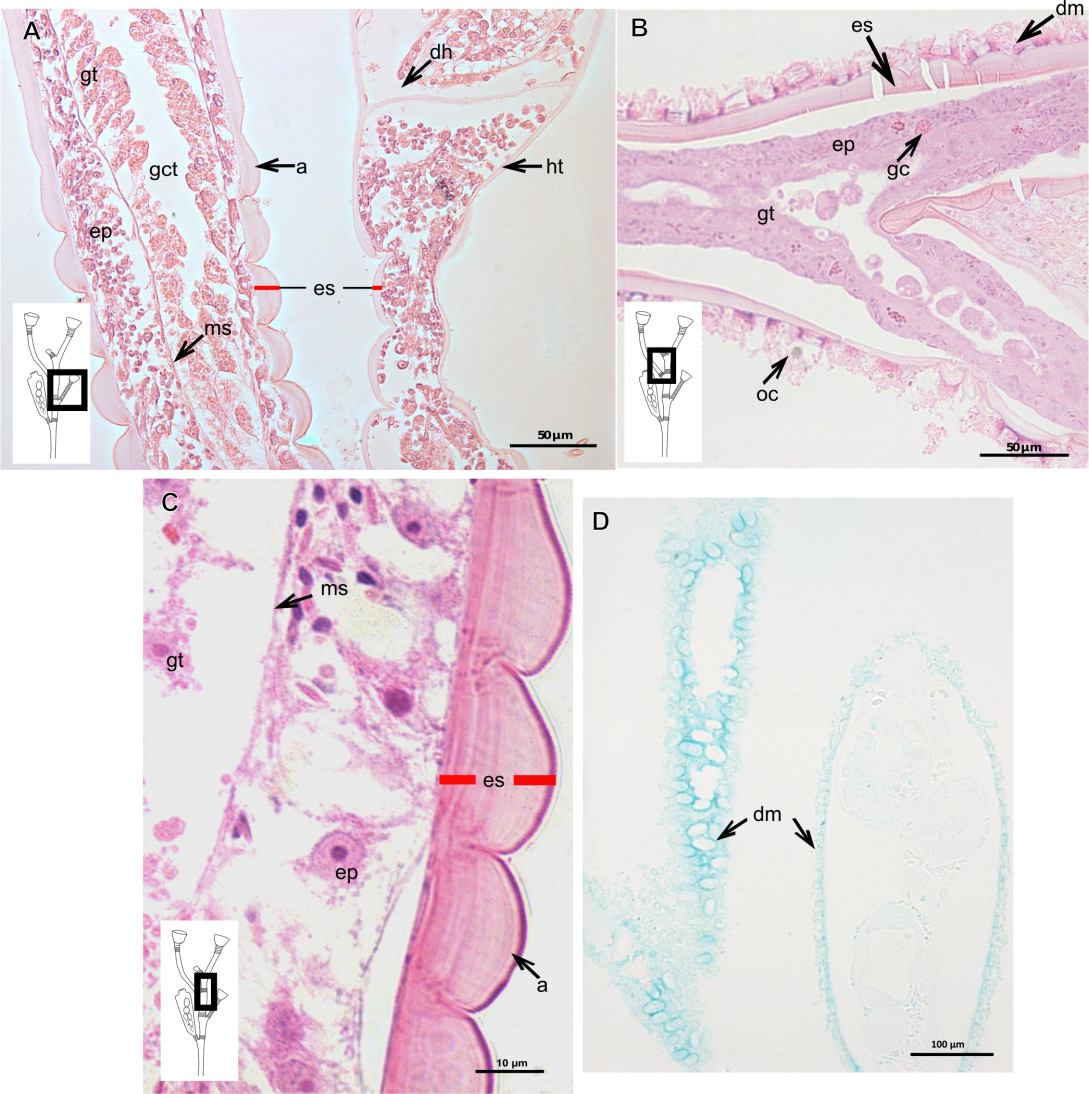
Exoskeletal structure of *Obelia dichotoma* (Linnaeus, 1758). (A)–(C) Stained with HE; (A) Exoskeleton of hydrocaulus and hydranth; (B) exoskeleton of the hydrocaulus; (C) exoskeletal layer in the hydrocaulus; (D) general exoskeletal structure with outermost cover and associated diatoms, stained with AB. Red line indicates the exoskeleton (=perisarc). Abbreviations: a, annulation; dh, diaphragm; dm, diatoms; ep, epidermis; es, exoskeleton; gc, glandular cell; gct, gastrovascular cavity; gt, gastrodermis; ht, hydrotheca; ms, mesoglea oc, outermost cover.

**Figure 21:**
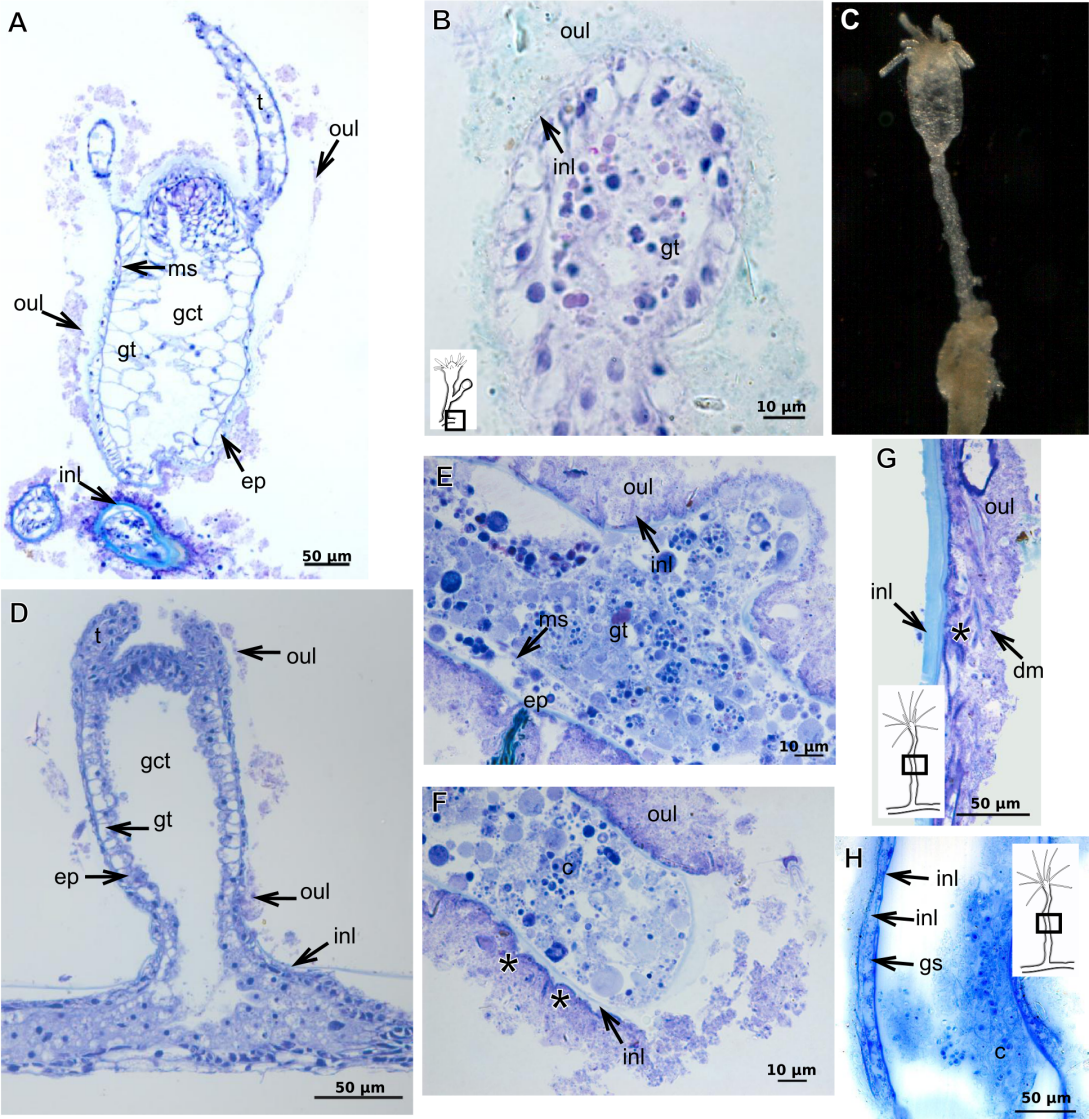
Development of the exoskeleton. (A)–(C) *Bimeria vestita* Wright, 1859. (A) Developing polyp in culture with filtered water of, stained with TB; (B) developing stolon, stained with AB + PAS + H; (C) external view of a polyp in culture with unfiltered water; (D)–(F) *Bougainvillia muscus* (Allman, 1863). (D)–(F) Stained with TB; (D) developing stolonal hydranth; (E) stolon of developing hydranth; (F) development of stolon of the hydrorhiza; (G)–(H) *Parawrightia robusta* Warren, 1907. (G) Hydrocauline exoskeleton of specimens maintained in culture conditions (unfiltered water), stained with AB; (H) hydrocauline exoskeleton of specimens maintained in culture conditions (unfiltered water), stained with HgBpB. Asterisk indicates “perisarc extensions.” Abbreviations: dm, diatoms; ep, epidermis; gct, gastrovascular cavity; gs, secretory granules; gt, gastrodermis; inl, inner layer; ms, mesoglea; oul, outer layer.

**Figure 22:**
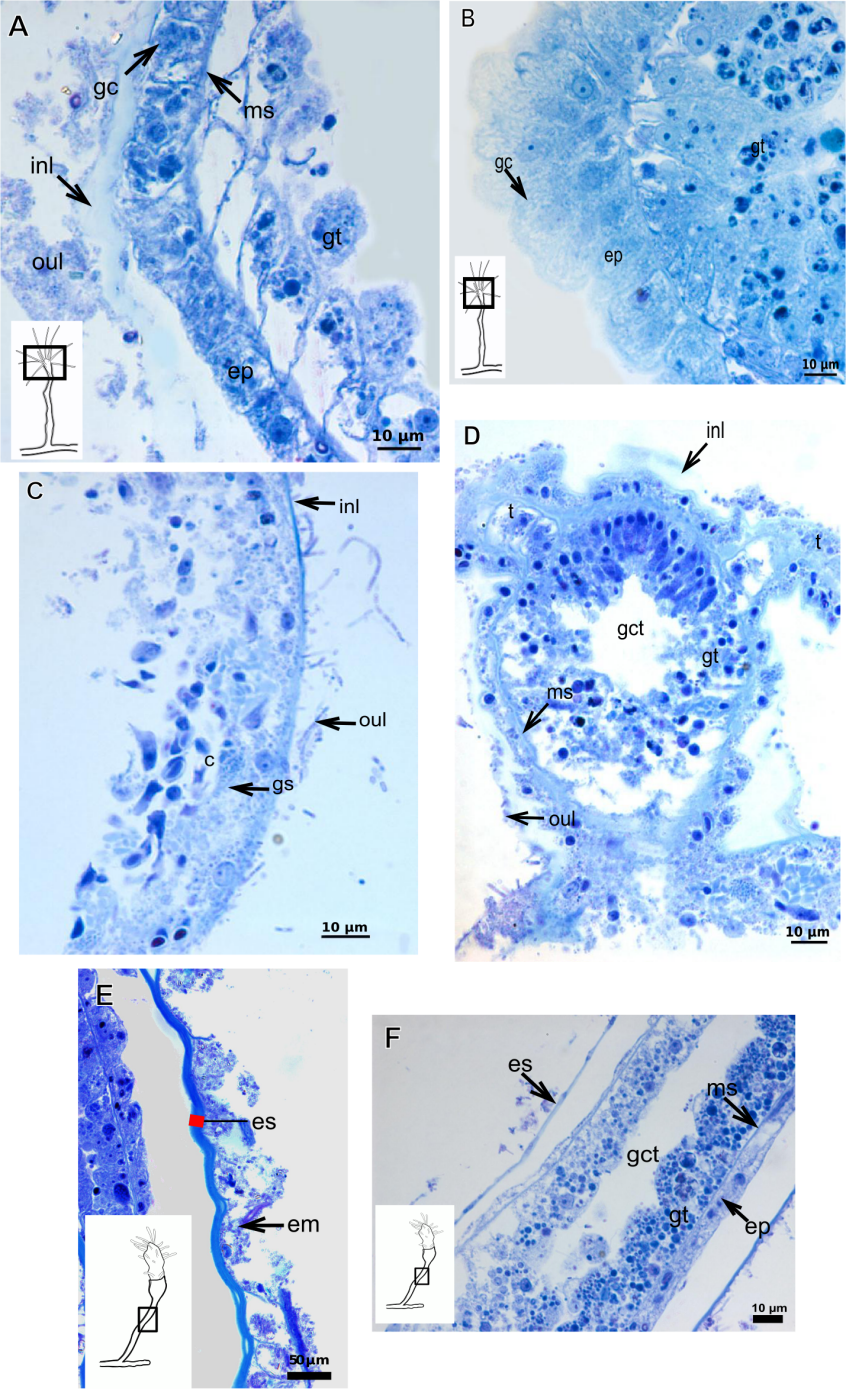
Development of the exoskeleton. (A)–(B) *Parawrightia robusta* Warren, 1907. (A)–(B) Glandular cells in the epidermis of the hydranth and developing polyp, stained with TB. (C)–(D) *Leuckartiara* cf. *octona* (Fleming, 1823), stained with TB. (E)–(F) *Turritopsis* sp. (E)–(F) stained with TB, (E) epidermis; (F) exoskeletal and coenosarc details of the hydrocaulus in a developing polyp. Red line indicates the inner layer of the exoskeleton. Abbreviations: c, coenosarc; em, external material; ep, epidermis; es, exoskeleton; gc, glandular cells, gct, gastrovascular cavity; gt, gastrodermis; inl, inner layer; ms, mesoglea; oul, outer layer.

***Orthopyxis sargassicola*** **(Nutting, 1915)**

Exoskeleton corneous (chitin-protein), rigid and laminated, moderately thick (Table 5), continuous from hydrorhiza to hydranth, annulated or sinuous throughout or only at proximal and distal ends of polyp (Figs. 19A–19D). Distal region of hydrocaulus with spherule (=subhydrothecal spherule), with basal annular thickening immediately above spherule (Fig. 19B). Contracted hydranth fully covered by exoskeleton (=hydrotheca), with cusps at border (Fig. 19B). Gonophore covered by exoskeleton (=gonotheca), with marked annulations (Fig. 19E). Some regions of polyp, especially hydrocaulus base and mid-part (Figs. 19C and 19D), with thin outer covering and with associated diatoms (Fig. 19F). Outer covering stains weakly with PAS and AB (Table 3).

**Table:** 

**Family Obeliidae Maronna et al., 2016**
***Obelia dichotoma* (Linnaeus, 1758)**

Exoskeleton corneous (chitin-protein), laminated, moderately thick (Table 5), continuous from hydrorhiza to hydranth, annulated on internodes and origin of side-branches (Figs. 20A and 20B). Exoskeleton with oblique diaphragm and smooth rim at distal region of hydrocaulus (Fig. 20A), entirely covering hydranth when retracted (=hydrotheca) and gonophore (=gonotheca; Fig. 16B). Exoskeleton of specimens from USA with vertical epidermis-parallel marks (Fig. 20C). Exoskeleton of specimens from Pará and Slovenia with diatoms attached, forming an outer covering mainly at hydrorhiza, with rigid appearance (Figs. 16B and 20B). Diatoms stain with PAS and intensely with AB (Fig. 20D).

### Description of hydroidolinan exoskeleton under culture conditions

Polyps of Bougainvilliidae *B. vestita*, *B. muscus* and *P. robusta*, and Pandeidae *L.* cf. *octona* maintained in culture with both filtered and unfiltered seawater developed a bilayered exoskeleton. The inner layer of *B. vestita* (Fig. 21A) is thinner at apical hydranth region close to tentacular whorl and at tentacular base (Fig. 21A), weakly stained with HgBpB and NYS compared to hydrorhizal and hydrocauline regions. The hydrorhiza has a thin perisarc and thick exosarc (Fig. 21B). The exoskeleton of a new hydranth is not detectable under the stereomicroscope (Fig. 21C), requiring histological preparations for detection. On the contrary, in *B. muscus* the exoskeleton is clearly seen at low power magnification, even in polyps 240 µm in height (Fig. 21D). *B. vestita* and *B. muscus* growing tips of the developing stolon and hydranth with ‘non-rigid’ inner layer (Figs. 21E and 21F), and outer layer encrusted with little external material. Inner layer of *P. robusta* is thinner, not rigid, with vertical divisions in some regions of hydrocaulus (Fig. 21G), and positive for HgBpB (Fig. 21H). Hydranth with thin exoskeleton (Fig. 22A) and developing polyps with granules in epidermal glandular cells (Figs. 22A and 22B). Outer layer of *L.* cf. *octona* with slightly different staining intensity with TB and AB compared to material developed under natural conditions. Exoskeleton at growing tips of the developing polyp of *L.* cf. *octona* with single “non-rigid” layer, encrusted with external material (Figs. 22C and 22D).

Polyps of *Turritopsis* sp. maintained in culture with unfiltered seawater developed the outer covering over exoskeleton but without encrusted material attached (Fig. 22E); polyps maintained in culture with filtered seawater did not developed an outer covering (developing polyp 2.1 µm) (Fig. 22F), and with affinity for PAS but not for AB, suggesting only AP present. Therefore, we assume this outer covering is not equivalent to the outer layer present in Bougainvilliidae. Polyps of *Clytia* sp. maintained in culture developed thin, AB-negative exoskeleton.

## Discussion

### Features of epithelia and their cells

Epidermal I-cells at the base of the hydrocaulus of *Turritopsis* sp., *L.* cf. *octona*, *H. bermudense* can be differentiated into nematocysts or glandular cells, the latter participating in the production of different substances forming the exoskeleton. This indicates the importance of these cells for both cnidogenesis and skeletogenesis, a hypothesis to be tested in other hydrozoan taxa.

The three types of epidermal glandular cells (vacuolated, granulated and mucous) were more abundant in developing polyps in the majority of the studied species of Bougainvilliidae, but were not observed in polyps of *D. conferta* and *P. disticha*. Previous histochemical tests indicated that the gastrodermal glandular cells of the hypostome continually produce and contain GAGs (*Syncoryne tenella* (Wineera, 1972), accepted as *Coryne eximia* Allman, 1859 (Schuchert, 2001); *Hydra* (Wood, 1979); MA Mendoza-Becerril, pers. obs., 2013); these substances may correspond to enzymes (Cowden, 1965). This is a different condition from the GAGs of the exoskeleton, which are not produced continuously based on our results and these are produced by epidermal mucous glandular cells.

Different enzymatic types and activities are specific for each function and region of the polyps, e.g., the enzyme acid phosphatase has been recorded in species of Leptothecata and its concentration has been associated with morphological variation of the species (Östman, 1982). Other important enzymes participating in exoskeleton formation have been recorded in several hydrozoans (Mendoza-Becerril et al., 2016). Chitin synthetase (Chs) is found in *Hydractinia echinata* (Mali et al., 2004). Chitinase is restricted to the gastrodermis of the hydrocaulus and absent in the epidermis and tentacles of *Podocoryna carnea* (accepted as *Hydractinia carnea*) and *Hydra attenuata*(accepted as *Hydra circumcincta*) (Klug et al., 1984). Phenoloxidase, produced in epidermal cells of *Laomedea flexuosa*, is involved in cross-linking of perisarc components (Knight, 1970; Kossevitch, Herrmann & Berking, 2001).

Our results corroborate the hypothesis that the coenosarc does not have a fixed composition of cell types. During development, different types of cells constantly migrate from specific areas of cell differentiation and proliferation to their final location (Chapman, 1974; Thomas & Edwards, 1991; Kosevich, 2013). Thus, cell action depends on a definite sequence of events, including cell multiplication, cell differentiation, and cell migration (see Kosevich, 2013; for *Gonothyraea loveni*). Also, the different epidermal glandular cells involved in exoskeletal development can change the type and secretion of one or several chemical components as the polyp grows (Kosevich, 2013).

**Figure 23:**
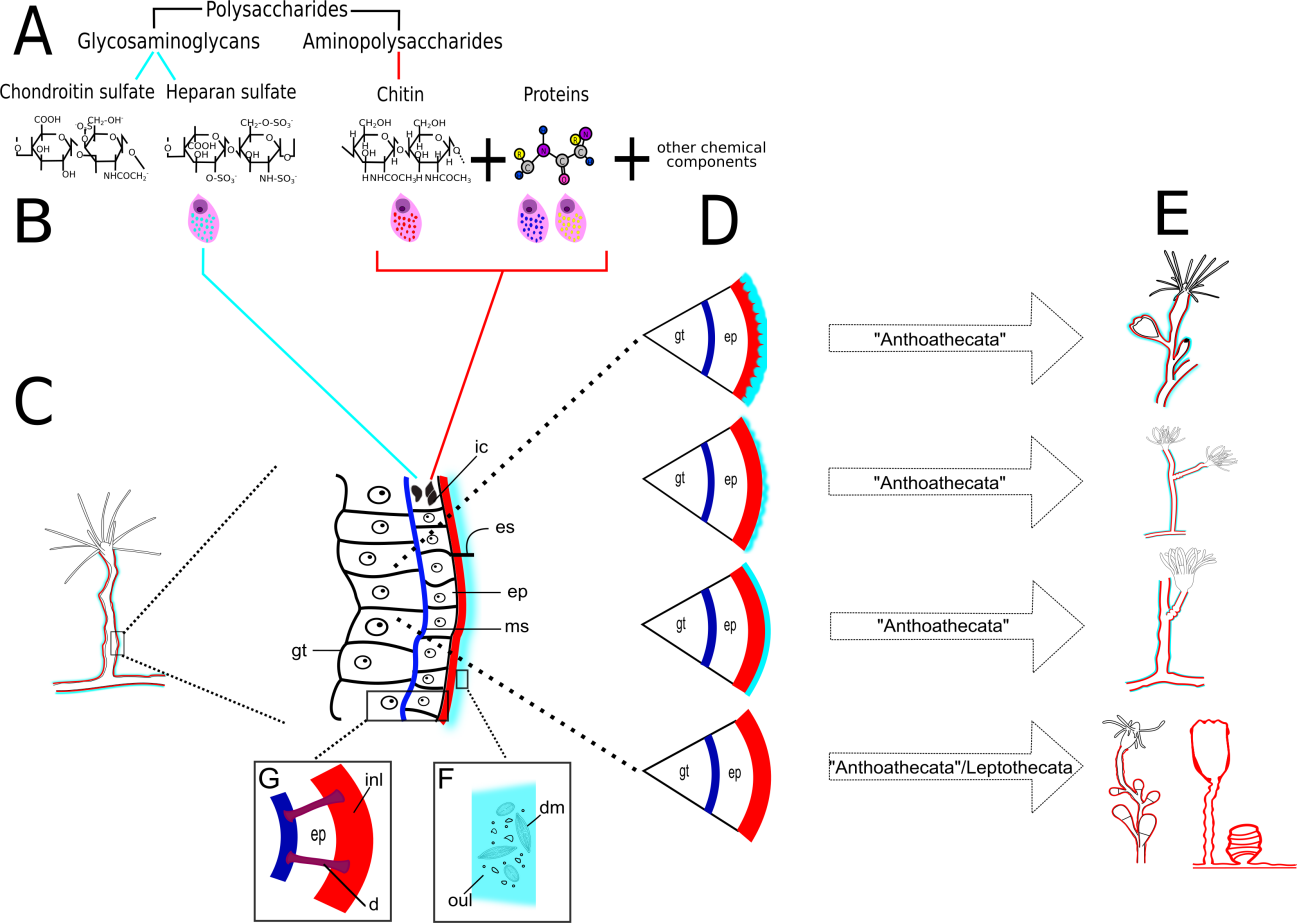
Schematic drawing of different chemical and structural types of the exoskeleton in the Hydroidolina. (A) Chemical components; (B) scheme of glandular and interstitial cells; (C) coenosarc and exoskeleton; (D) structural types of the exoskeleton; (E) exoskeleton extension in polyps of “Anthoathecata” and Leptothecata; (F) outer layer encrusted with inorganic and organic material; (G) desmocytes connecting inner layer with mesoglea. Cyan-blue line indicates the outer layer of the exoskeleton, red line indicates the inner layer of the exoskeleton. Abbreviations: d, desmocyte; dm, diatoms; ep, epidermis; es, exoskeleton; gt, gastrodermis; ic, interstitial cells; inl, inner layer; ms, mesoglea; oul, outer layer.

### Exoskeleton organization and chemical composition

Our findings confirm that polysaccharides are the basic and predominant chemical component of the exoskeleton (Knight, 1970; Kossevitch, Herrmann & Berking, 2001; Mendoza-Becerril et al., 2016) (Figs. 23A and 23B). These polysaccharides can combine with units of amino-sugars, forming aminopolysaccharides (AP) (Ruiz-Herrera & Ortiz-Castellanos, 2010) (PAS-positive); amino-sugars and hexuronic acid, forming glycosaminoglycans (GAGs) (Frazier et al., 2008; Yamada, Sugahara & Özbek, 2011) (PAS-positive and AB-positive); or only with structural proteins (glycoproteins) (BeMiller, 2008) (HgBpB and NYS-positive) (Table 3). The predominant exoskeletal component are AP, in the form of chitin (Mendoza-Becerril et al., 2016), but there are variations in the chemical composition and in the physical properties related to adaptations to particular conditions and physiological changes in the organism, such as the tanning process (Chapman, 1973).

We documented two types of structural exoskeleton, (a) the bilayered exoskeleton formed by an inner layer of perisarc surrounding the coenosarc and covered by an outer layer of exosarc in contact with the environment, and (b) a single coriaceous exoskeleton formed exclusively by the perisarc (Figs. 23C and 23D). The perisarc and exosarc vary in their chemical composition (AP or GAGs; Table 4), texture (Fig. 23D), thickness, extension and coverage of different regions of the colony (Fig. 23E). A thick exosarc is generally derived from the aggregation of extraneous inorganic (sand and mud grains) and organic materials (tests of radial centric and araphid pinnate diatoms) (Mendoza-Becerril et al., 2016). These extraneous materials lend a rigid granular appearance to the exoskeleton of Bougainvilliidae and Pandeidae (Fig. 23F).

All the species of Bougainvilliidae that we studied have a bilayered exoskeleton, with the possible exception of *P. michaeli*. For this species we found a discontinuous thin layer in its hydrorhiza and hydrocaulus. This thin layer could correspond to exosarc, contradicting the descriptions of *P. michaeli* stating that the “pseudohydrotheca” (=exosarc on the hydranth) is absent in this species (e.g. Schuchert, 2007).

Gonophores of the bougainvilliids studied (except *P. michaeli*) are completely enclosed by a bilayered exoskeleton, even in the species that have been described with an exoskeleton restricted to the gonophore pedicels, such as *G. annulata* (Nutting, 1901). As it is very thin and flexible, almost imperceptible in some cases when specimens are examined entire, the exoskeleton completely surrounding the gonophore is described in the literature as a filmy perisarc, loose filmy perisarc, or thin perisarc membrane (Schuchert, 2007).

Epidermal glandular cells of the colonies of Bougainvilliidae (*B. vestita, B. muscus,* and *P. robusta*) and Pandeidae (*L.* cf. *octona*), forming the molecular matrix (MM), apparently are differentiated at the developing border of the free stolons/branch, hydrorhiza, side-branch, stolonal and terminal hydranths. Developing extremities of growing polyps and hydranths of some bougainvilliids were covered by a “non-rigid” layer of GAGs, while regions near their origin were covered by a “non-rigid” exoskeleton formed by an exosarc, constituted predominantly by GAGs, and a perisarc with AP and proteins. This “non-rigid” layer may correspond to MM and to the cuticle described by Wineera (1972), possibly being involved in the process of formation of a rigid, bilayered exoskeleton (perisarc and/or exosarc). Particles and thin filaments present in the exoskeleton would serve to harden the structure.

The exosarc is produced first, and may be an important step in the formation of the rigid perisarc. The exosarc could interact with other molecules (e.g., AP, structural proteins) functioning as a single layer in developing polyps. This hypothesis is supported by the presence of AB-positive granules in the epidermal glandular cells at the base of the hydrocaulus of *G. franciscana*, and of TB- and PAS-positive granules in the skeletal outer layer and epidermal glandular cells of the developing hydranth of *L.* cf. *octona*. The MM with acid polysaccharides is an important element in the mineralization process in the stony coral *Mycetophyllia reesi* (Goldberg, 2001).

Variations in staining intensity suggest the presence of different concentrations of the chemical components, depending on the developmental stage of the polyps. The presence of GAGs in the perisarc of *G. annulata*, *E. carneum*, and *C. gracilis* indicates that acidic GAGs are trapped within the inner layer, maybe sclerotized in the presence of proteins. The mix of GAGs and proteins (glycoproteins) is common in mollusk shells (Marxen et al., 1998), functioning as possible calcium-binding sites or playing a role in the nucleation and growth inhibition of the mineral (Marin et al., 1996).

A perisarc was formed in all species studied, although the perisarc was sometimes weakly stained with PAS and intensely with NYS in bougainvilliids (especially *B. rugosa*). Chitin reacts negatively with PAS, but positively in protein tests if it is present as protein complexes (Pearse, 1985). Chitin does not occur in its pure form in nature, but always mixed with protein and/or other chemical substances (Pearse, 1985). However, the absence of chitin in a tissue sample does not necessarily indicate that the species is not able to produce chitin, because it may be modified (deacetylated or sulfated) and undetectable by classical histochemical methods—only finding chitin synthase activity or expression of genes related to chitin synthases would corroborate the presence of chitin (Wagner, 1994). Indeed, chitin has been found in the perisarc as part of the leptothecatan exoskeleton (e.g., *L. flexuosa*, (Knight, 1970); *Aglaophenia latirostris*, (Hwang et al., 2013)).

The perisarc has been described as multilayered in the hydrorhiza and hydrocaulus (Wineera, 1972), although the laminae can be either continuous or discontinuous (Fig. 3H). The laminated perisarc (Fig. 3B) has multiple discontinuous fibers, extending in parallel through the length of the thick, rigid perisarc. In addition, the laminated perisarc can be reticulated, with grooves perpendicular to the fibers (Fig. 3B). The laminated and reticulated appearance is most likely a consequence of different degrees of stabilization and hardening during the polymerization (Berrill, 1949) or of sclerotization process (Knight, 1970), and it is more frequent in developed polyps because of the higher concentration and interaction of molecules incorporated into the MM (Congdon, 1906). However, our understanding of the chemistry of exoskeleton sclerotization has not much improved since Knight (1968), who suggested that “tanning cells” (observed in the Leptothecate *L. flexuosa*) are essential to the process (Knight, 1970). Therefore, many important questions remain unanswered, especially regarding the precise regional and temporal regulation of the various steps in the process.

We observed secretory granules (HgBpB-positive) scattered in the hydrocaulus of developing polyps of *P. robusta,* and glandular cells (PAS-positive) in new side-branches of *P. disticha,* as well as in developing polyps of *Turritopsis* sp. and the hydrocaulus of *Clytia* sp. The presence of secretory granules and glandular cells, and their reaction to different chemical tests could be indicative of a sclerotization process in “Anthoathecata,” although specific studies are necessary to test this hypothesis.

The “non-rigid” exosarc is detected by AB pH 2.5, suggesting a chemical composition of GAGs (carboxylic groups). However, only some species (e.g., *B. rugosa, L.* cf. *octona*) have epidermal glandular cells with an affinity for AB. Therefore, the origin of the exoskeletal acidic GAGs is not clear. Some data support different hypotheses regarding their origin and variable composition in chemical specific groups, such as the reactivity of AB pH 2.5 with GAGs influenced by specific GAG-associated properties (structure, purity, and other factors), and the failure to detect them among major heparin and unsulfated types of GAGs (Frazier et al., 2008).

Developing polyps have only an exosarc, suggesting that this is the first layer in the skeletal ontogenesis. Subsequently, epidermal cells differentiate, producing other specialized glandular cells and therefore changing the nature of the secreted compounds over time. Such changes may have prevented us from observing mucous glandular cells in some species, which would be capable of rapidly eliminating their secretions and developing into different cell types, as in *Hydra pseudoligactis* (accepted as *Hydra canadensis*), a species with epidermal cells that release acid GAGs (Burnett & Lambruschi, 1973).

The thin, discontinuous outer covering observed in the exoskeleton of athecate *Turritopsis* sp. and some leptothecates (*O. dichotoma* and *O. sargassicola*), with an affinity for AB, has adhered inorganic and/or organic external material. However, this covering is probably not part of the exoskeleton and not equivalent to the exosarc of bougainvilliids and some other members of “Anthoathecata,” because we have not observed a MM in the species with discontinuous outer covering throughout the polyp, and, at least in the case of *Turritopsis* sp., it does not develop when the polyp is maintained in filtered seawater. The outer covering is possibly formed by exogenous substances, particles or diatoms. For example, the diatoms secrete acid sugars in the form of uronic acids and sulfated sugars (Staats et al., 1999) and therefore positive to AB, thus our results indicate that leptothecate polyps are incapable of producing GAGs independently.

In most species we observed that the exoskeleton was laid down even when the colonies were maintained with filtered seawater, therefore suggesting its secretion is genetically encoded and innate (a putative MSS), and does not depend on age or environmental conditions. Exoskeleton thickness, especially of the exosarc, depends on the quantity and type of extraneous material (organic or inorganic) available under natural conditions (Rees, 1956; Schuchert, 2007; Mendoza-Becerril et al., 2016) and the exoskeletal morphology can be modified by environmental conditions (Murdock, 1976; Hughes, 1980) using any source of external material, even agglutinating particles egested by the polyp. Some species have a polymorphic expression of the exosarc in at least some species of *Garveia*. This was observed when comparing specimens from different environments. Similarly, it has been observed in other Hydroidolina (Rees, 1956; Murdock, 1976; Hughes, 1980).

Different levels of contraction were observed in living and fixed polyps. A contracted hydranth of some species, such as *B. muscus* and *G. franciscana*, appears to be fully covered by the bilayered exoskeleton, even though their exoskeleton extends only from the hydrorhiza to the tentacular base of the hydranth; while other species, such as *G. nutans* and *P. robusta*, appear to be covered up to the whorl of tentacles, as delimited by a fold just below that, although the extended body clearly has free tentacles. Species such as *B. vestita* may have the hydranth completely covered by the exoskeleton even when fully extended, but, without a detailed analysis, may appear to have exoskeleton coverage similar to contracted hydranths of *B. muscus* or *G. franciscana*.

The thickness of the exoskeletal layer (Table 5) varies intraspecifically in some “Filifera” from different locations (e.g., Table 7 and *G. franciscana*, Vervoort, 1964). Consequently, this structural variation may lead to misidentifications of the species, especially when other diagnostic characters are absent (e.g., reproductive structures), and should be used cautiously as diagnostic for the taxonomy of groups such as Pandeidae and Bougainvilliidae (Millard, 1975; Calder, 1988). Nevertheless, the variation in thickness of the chitin-protein exoskeleton is considered a useful diagnostic character for some families of Leptothecata (e.g., Clytiidae, Cunha, Genzano & Marques, 2015).

Desmocytes (Fig. 23G) are specialized cells that are found along the upright hydrocauline coenosarc of the polyps and side branches of colonies of Bougainvilliidae, Eudendriidae, Pandeidae, and Haleciidae, among the species studied. They are characterized by a dense accumulation of chitin and protein filaments (Knight, 1970; Chapman, 1974), and therefore have a high affinity for PAS, HgBpB and NYS. These filaments aggregate into dense rods and reach the exoskeletal perisarc at the apical end of the desmocyte (Fig. 23G), and form rigid connections with the mesoglea at the basal end of the desmocyte (Fig. 23G).

Desmocytes (also termed “rivets” by Knight, 1970 and “anchors” by Buss, Anderson & Bolton, 2013) function similarly to other anchoring devices in Hydrozoa (e.g., Cordylophoridae *Cordylophora caspia*, Marcum & Diehl, 1978); Lafoeidae *Lafoea benthophila,* (Antsulevich & Vervoort, 1993); Zancleidae *Zanclea margarita*, (Pantos & Hoegh-Guldberg, 2011); Hydractiniidae *Podocoryna carnea*, (Buss, Anderson & Bolton, 2013); Obeliidae *L. flexuosa*, (Knight, 1970), Scyphozoa (Ulmariidae *Aurelia* sp., (Lesh-Laurie & Suchy, 1991)), and Cubozoa (Carybdeidae *Carybdea* sp., (Mendoza-Becerril et al., 2016)). However, these cells show structural differences that may be related to the pattern of exoskeleton extension, colony growth, and symbiotic relationships (Chapman, 1974), which could also serve to explain the apparent absence of these cells in some Hydroidolina we have studied. We suggest that, comparing to the other anchoring structures observed, “perisarc extensions” are a still-undescribed type of anchoring system, acting in the adherence between the perisarc and exosarc. The “perisarc extensions” increase the rigidity of the exoskeleton, and are present along the upright hydrocaulus. These structures were also described as “lamellar membranes” in the bougainvilliid *Garveia grisea* (Schuchert, 2007).

In conclusion, our study added to the knowledge of the hydrozoan exoskeleton, but also left unanswered several questions on its structure and chemical composition: which specific components are present within the exoskeleton (e.g., glycoproteins, proteoglycans and hexuronic acids, more specifically, chondroitin sulfate and heparan sulfate)? What is the ratio of the different chemical components and what are their chemical interactions? What are the biomechanical properties related to the different types of exoskeletons and their biological consequences? Further investigations applying immunohistochemistry (e.g., to identify the type of GAGs), confocal microscopy (e.g., using congo red as a fluorescence marker for chitin), and transmission electron microscopy and X-ray diffraction may help to answer these questions.

## Additional Information and Declarations

### Data Availability

The following information was supplied regarding data availability: The physical specimens are available long term, but as there isn’t anyone dedicated exclusively to their care, they need to be requested in advance from Dr. Antonio C. Marques at Centro de Biologia Marinha (CEBIMar), Universidade de Sao Paulo.
http://www.usp.br/cbm
Phone (CEBIMar): (55 12) 3862 8422 E-mail: mailto:cebimar@usp.br Mail address: Depto Zoologia, Instituto de Biociencias Universidade de Sao Paulo Rua Matao, Trav. 14, 101, 05508-090, Sao Paulo, Brazil Phone (Zoologia): (55 11) 30917530 Fax: (55 11) 30917802 Laboratory of Marine Evolution, Institute of Biosciences, University of São Paulo, Brazil. LEM-IBUSP_7, LEM-IBUSP_1, UNMdP Hd3-38, LEM-IBUSP_2, LEM-IBUSP_3, UNMdP Hd11-128, LEM-IBUSP_4, LEM-IBUSP_5, LEM-IBUSP_6, LEM-IBUSP_8 National Museum of Natural History, Smithsonian Institution (USNM) USNM 43497 http://n2t.net/ark:/65665/3c1ac9b93-e32b-4ff5-a01e-0f783ac3299a
USNM 20234 http://n2t.net/ark:/65665/3140bc468-acad-4cea-93ba-7d1e841e096f
USNM 43967 http://n2t.net/ark:/65665/3663195dc-c052-430e-9984-65378e40368a
USNM 71026 http://n2t.net/ark:/65665/33d78f0a1-a446-4839-99da-2077fd312e55
USNM 43496 http://n2t.net/ark:/65665/3ba9e0775-542d-4fb5-be63-2ab4b5dfaf28
USNM 89229 http://n2t.net/ark:/65665/39a3893eb-6e84-4222-957d-7e691bd17bbe
USNM 43330 http://n2t.net/ark:/65665/39524587f-77b1-4fb1-a068-a964705b2ba6
USNM 29449 http://n2t.net/ark:/65665/30d85ddd6-543d-4278-9d19-d8574cc2ab90
USNM 42339 http://n2t.net/ark:/65665/3508d03d1-aca2-4918-8538-be93c9734b38
Museum of Zoology, University of São Paulo (MZUSP) MZUSP 5201, MZUSP 4217, MZUSP 4332, MZUSP 1832, MZUSP 003390, MZUSP 4379, MZUSP 1673, MZUSP 003391, MZUSP 4210, MZUSP 1740, MZUSP 3371, MZUSP 3369.
